# Proliferating cell nuclear antigen inhibitors block distinct stages of herpes simplex virus infection

**DOI:** 10.1371/journal.ppat.1011539

**Published:** 2023-07-24

**Authors:** Jessica E. Packard, Maya R. Williams, Daniel P. Fromuth, Jill A. Dembowski

**Affiliations:** Department of Biological Sciences, Duquesne University, Pittsburgh, Pennsylvania, United States of America; National Cancer Institute, UNITED STATES

## Abstract

Proliferating cell nuclear antigen (PCNA) forms a homotrimer that encircles replicating DNA and is bound by DNA polymerases to add processivity to cellular DNA synthesis. In addition, PCNA acts as a scaffold to recruit DNA repair and chromatin remodeling proteins to replicating DNA via its interdomain connecting loop (IDCL). Despite encoding a DNA polymerase processivity factor UL42, it was previously found that PCNA associates with herpes simplex virus type 1 (HSV-1) replication forks and is necessary for productive HSV-1 infection. To define the role that PCNA plays during viral DNA replication or a replication-coupled process, we investigated the effects that two mechanistically distinct PCNA inhibitors, PCNA-I1 and T2AA, have on the HSV-1 infectious cycle. PCNA-I1 binds at the interface between PCNA monomers, stabilizes the homotrimer, and may interfere with protein-protein interactions. T2AA inhibits select protein-protein interactions within the PCNA IDCL. Here we demonstrate that PCNA-I1 treatment results in reduced HSV-1 DNA replication, late gene expression, and virus production, while T2AA treatment results in reduced late viral gene expression and infectious virus production. To pinpoint the mechanisms by which PCNA inhibitors affect viral processes and protein recruitment to replicated viral DNA, we performed accelerated native isolation of proteins on nascent DNA (aniPOND). Results indicate that T2AA inhibits recruitment of the viral uracil glycosylase UL2 and transcription regulatory factors to viral DNA, likely leading to a defect in viral base excision repair and the observed defect in late viral gene expression and infectious virus production. In addition, PCNA-I1 treatment results in decreased association of the viral DNA polymerase UL30 and known PCNA-interacting proteins with viral DNA, consistent with the observed block in viral DNA replication and subsequent processes. Together, we conclude that inhibitors of cellular PCNA block recruitment of key viral and cellular factors to viral DNA to inhibit viral DNA synthesis and coupled processes.

## Author summary

The goal of this study was to determine what role PCNA associated with HSV-1 replication forks plays during viral infection. Using two commercially available PCNA inhibitors, we provide evidence that targeting specific functions of PCNA block HSV-1 DNA replication and subsequent steps in the infectious cycle. Although it was previously thought that viral factors alone facilitate the process of HSV-1 DNA replication, results presented here contradict this long-standing view. With the continued emergence of antiviral resistant HSV-1 variants, these results provide insight into how cellular proteins can be targeted for antiviral treatment.

## Introduction

Herpes simplex virus type 1 (HSV-1) is a ubiquitous pathogen that infects over half of the human population [1,2]. Lytic infection occurs in epithelial cells where the HSV-1 genome replicates within the nucleus. Upon entry into the nucleus, viral genes are transcribed by cellular RNA polymerase II through a temporal cascade of immediate early, early, and late genes [3–5]. The recruitment of transcription factors to immediate early gene promoters early during infection results in the expression of immediate early genes [6], including the major viral transcription factor ICP4 [7]. ICP4 recruits transcription factors to activate downstream early genes [8–12], which encode the viral replication proteins. Viral DNA replication and ICP4 promote transcription of late viral genes classified as leaky late (amplified with viral DNA replication) or true late (turned on after viral DNA replication) genes [13,14]. However, the mechanism by which DNA replication licenses true late gene expression is not known. Late genes encode viral structural proteins including glycoproteins, tegument proteins, and capsid proteins [13,15]. After primary infection in the epithelial cells, HSV-1 can establish latency in innervating sensory neurons for the lifetime of the host. Stress and other stimuli can cause HSV-1 to reenter the lytic cycle but the mechanisms of reactivation are not well understood [16,17].

Seven viral factors are necessary for replication of the ~152 kbp HSV-1 genome [18,19]. These include an origin binding protein (UL9), single stranded DNA binding protein (ICP8), helicase/primase complex (UL5, UL8, and UL52), DNA polymerase (UL30), and processivity factor (UL42). In addition to the viral replication machinery, proteomic approaches have identified several cellular factors that are enriched on viral replication forks and replicating viral DNA [20–22]. Cellular factors enriched on HSV-1 replication forks include topoisomerases, mismatch repair proteins, base excision repair factors, the MRN complex (NBS1, MRE11, and RAD50), replication factor C (RFC), and proliferating cell nuclear antigen (PCNA).

**Fig 1:**
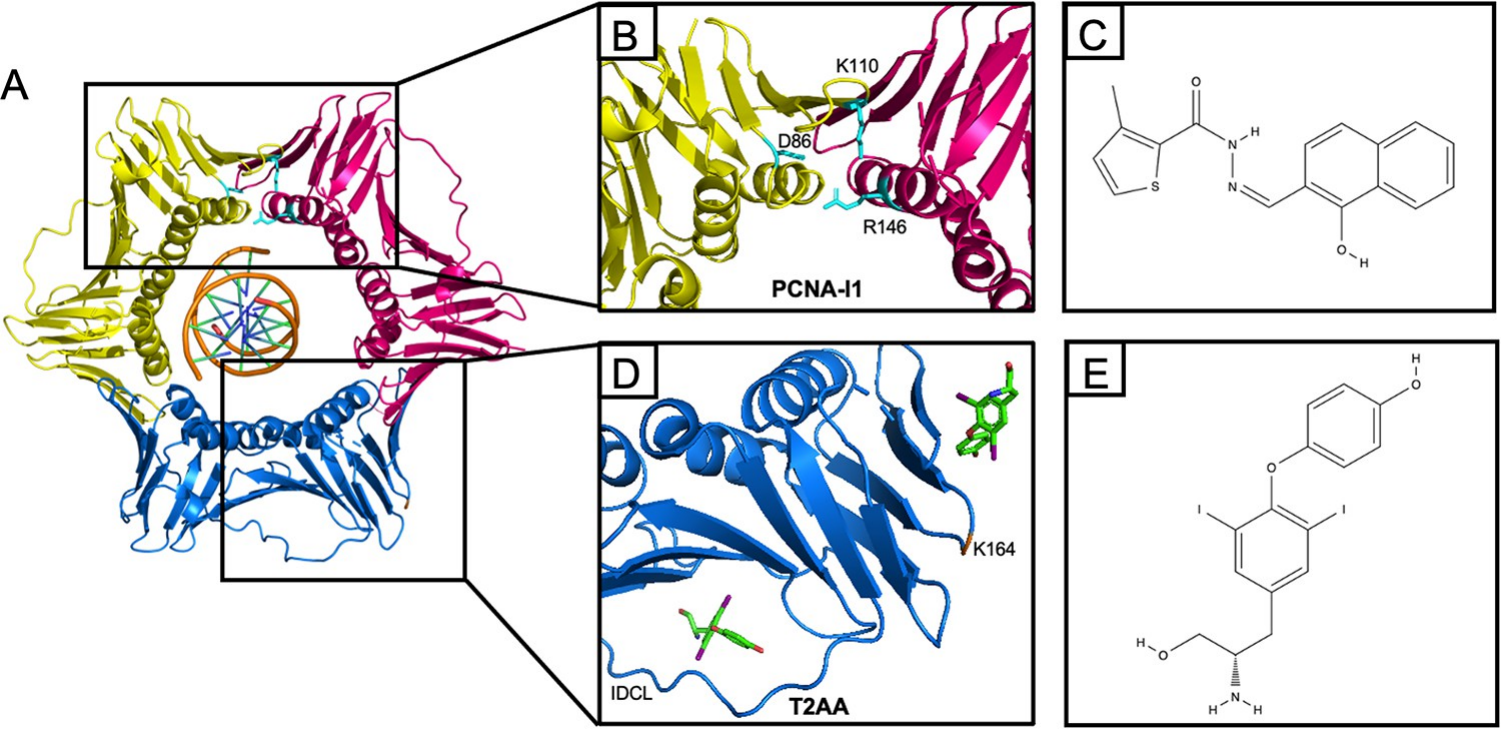
The structure of PCNA and the docking of small molecule inhibitors. A) The structure of the PCNA homotrimer encircling double stranded DNA (PDB file 6GIS) [79]. Black boxes highlight the areas where PCNA-I1 and T2AA bind. B) PCNA-I1 is predicted to bind to the interface between two PCNA monomers by molecular modeling [40]. Specific residues that are predicted to interact with PCNA-I1 are labeled in light blue. D86 of one PCNA monomer (pink) is predicted to bind to the PCNA-I1 inhibitor via a N-O hydrogen bond. Residue K110 of the same monomer (pink) is predicted to form a nonpolar bond via the aromatic rings of PCNA-I1. R146 on an adjacent PCNA monomer (yellow) is modeled to bind to PCNA-I1 via an O-N hydrogen bond. C) The chemical structure of PCNA-I1. D) Structure of T2AA bound to PCNA. It was found that two molecules of T2AA bind to a PCNA monomer (blue) (PDB file 3WGW) [45]. T2AA binds within the interdomain connecting loop where proteins containing the PIP-box motif interact. The second T2AA molecule binds to the PCNA monomer adjacent to K164 (residue labeled in orange). E) Chemical structure of T2AA. Images of PCNA created with PyMOL.

PCNA is an essential eukaryotic DNA replication protein that forms a homotrimer that encircles cellular DNA (Fig 1A) [23]. PCNA is loaded onto DNA by RFC and functions as a DNA sliding clamp for cellular DNA polymerases. In addition, PCNA can interact with specific factors involved in DNA repair, lagging strand synthesis, and chromatin assembly via the interdomain connecting loop (IDCL). PCNA is central to Okazaki fragment maturation [24], translesion synthesis [25–29], nucleotide excision repair [30], homologous recombination [31], and mismatch repair [23,32–34]. In addition, PCNA is post-translationally modified to regulate protein-protein interactions in response to DNA damage and the cell cycle [35]. Therefore, PCNA is a versatile and key cellular replication factor that coordinates DNA replication with other cellular processes.

Previous studies have demonstrated that PCNA is recruited to viral replication forks in a replication-dependent manner [21] and is necessary for efficient viral DNA replication in vivo [20,21,36]. As mentioned, HSV-1 encodes its own processivity factor, UL42 [37]. UL42 is structurally similar to PCNA despite sharing no sequence homology [38]. However, unlike other DNA polymerase processivity factors, UL42 functions as a monomer [38,39] to tether the HSV-1 DNA polymerase UL30 to viral DNA [37]. Perhaps PCNA carries out unique functions at viral replication forks that UL42 is unable to facilitate such as encircling DNA and/or recruiting replication, repair, or chromatin remodeling factors.

To investigate the involvement of PCNA in viral processes, HSV-1 infected cells were treated with two commercially available PCNA inhibitors that have different mechanisms of action. PCNA-I1 stabilizes the PCNA homotrimer (Fig 1B and 1C) [40,41] and reduces the repair of double strand breaks by homologous recombination and suppresses nucleotide excision repair [42]. T2AA inhibits interactions between proteins that contain a PCNA interacting protein (PIP)-Box motif and the PCNA IDCL or mono-ubiquitinated K164 (Fig 1D and 1E) [43–45]. This results in disruption of the interaction between PCNA and the cellular translesion polymerase η and inhibition of the cellular translesion synthesis pathway [43]. Testing the effects of these two inhibitors on HSV-1 infection could therefore pinpoint the functions of PCNA during HSV-1 infection.

Here we identified novel antiviral effects of PCNA inhibitors on HSV-1 infection. We found that PCNA-I1 and T2AA do not block early steps in viral infection including viral immediate early and early gene expression. T2AA treatment had little to no effect on viral DNA replication but caused a decrease in late viral gene expression and infectious virus production. PCNA-I1 treatment of infected cells resulted in a strong inhibition of viral DNA replication and late gene expression, resulting in significant defects in infectious virus production.

Because PCNA functions as a scaffold to recruit repair factors and polymerases to replicating cellular DNA, we hypothesized that PCNA inhibitors block protein recruitment to replicating HSV-1 DNA leading to defects in viral DNA replication, gene expression, and infectious virus production. We used accelerated native isolation of proteins on nascent DNA (aniPOND) to determine what effect each inhibitor has on protein recruitment to replicating viral DNA [11,20,21]. During PCNA-I1 treatment, several proteins that are enriched at viral replication forks were reduced on replicated viral DNA, with the most notable effect on the UL30 viral DNA polymerase. In addition, there was an increase in recruitment of Rad50 and Mre11 to viral DNA. Together, these results are consistent with the viral DNA replication defect observed in the presence of PCNA-I1. T2AA inhibition caused decreased association of the viral base excision repair factor UL2 and transcription regulatory factors including components of the host Integrator complex. Together, these defects likely contribute to the observed reduction in late viral gene expression and infectious virus production. The results presented here are consistent with a model whereby PCNA is present at viral replication forks and inhibition of viral infection with PCNA inhibitors blocks key aspects in viral DNA replication and coupled processes. Furthermore, these results reveal the potential to target PCNA or PCNA-interacting proteins for HSV-1 antiviral therapy.

## Results

### Optimization of conditions for PCNA-I1 and T2AA treatment of cells

**Fig 2:**
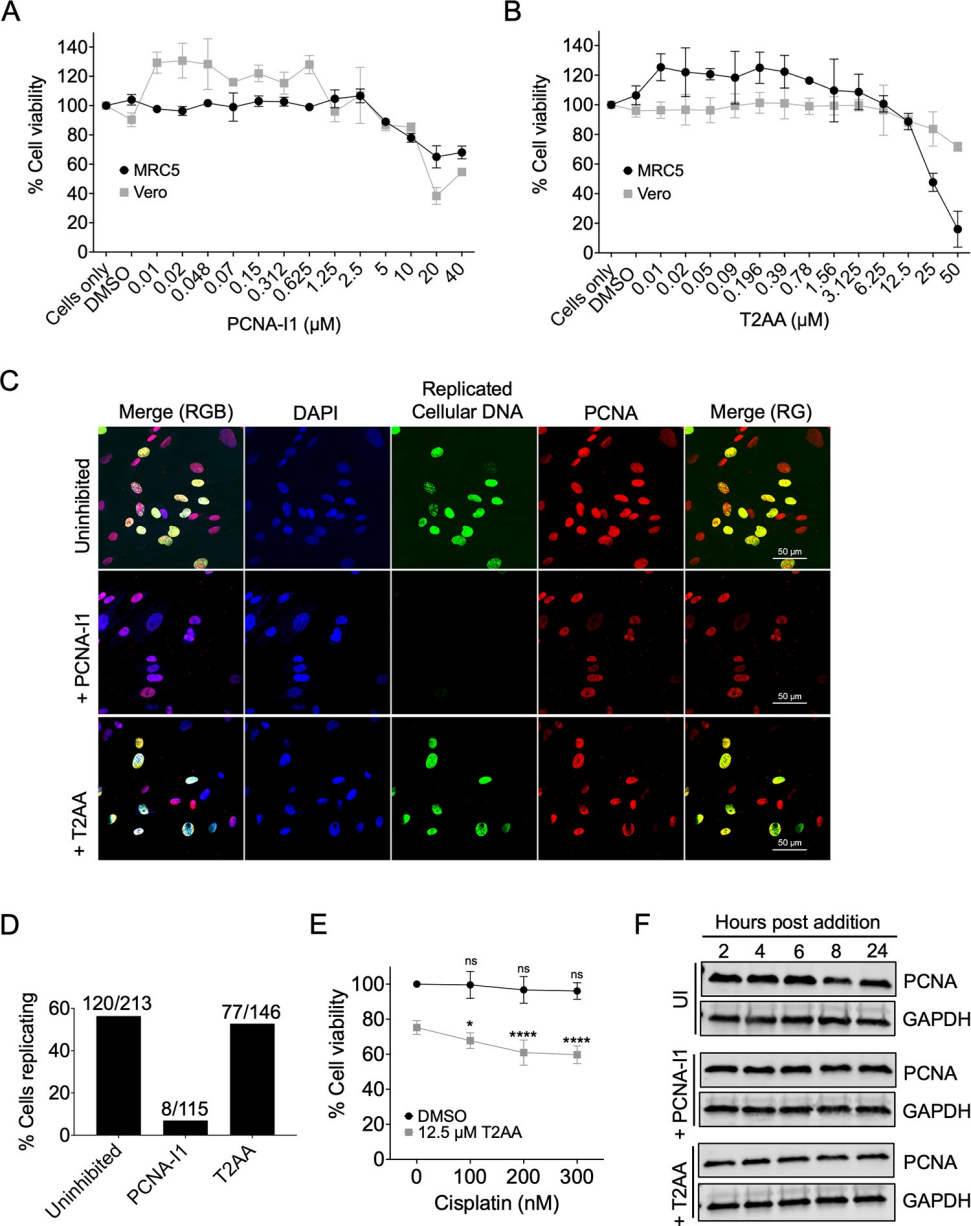
Effect of PCNA inhibitors on cell viability, DNA replication, and PCNA protein levels. A-B) The Cell Titer Glo Luminescent Cell Viability assay was carried out to measure the cytotoxic effects of PCNA-I1 (A) and T2AA (B) on MRC-5 and Vero cells. Cells were incubated in the presence of indicated concentrations of inhibitor for 24 hours before conducting the assay. Data represent the mean of biological triplicate experiments with standard deviation. Each biological replicate was determined as the mean of 3 technical replicates. C) Immunofluorescence images of MRC-5 cells that were plated at a low density and uninhibited or treated with 2.5 μM PCNA-I1 or 12.5 μM T2AA for 6 hours. After 4 hours, EdC was incorporated into replicating cellular DNA for two hours. Following fixation, nuclei were stained with Dapi, EdC-labeled DNA was tagged with Alexa Fluor 488, and PCNA was stained by immunofluorescence. Scale bars, 50 μm. D) The percentages of replicating cells after PCNA-I1 or T2AA inhibition were calculated from 10 images captured as in (C). The number of nuclei with EdC incorporation divided by total nuclei counted is indicated above each bar graph. E) The CellTiter-Glo Luminescent Cell Viability assay was carried out as in (B) in the presence of cisplatin. Cells were incubated in the presence of the indicated concentration of cisplatin and/or 12.5 μM T2AA for 24 hours before conducting the assay. Data represent the mean of biological triplicate experiments with standard deviation. Each biological replicate is an average of three technical replicates. One-way ANOVA with the Dunnett’s multiple comparisons test was performed to compare cell viability in the presence of 100, 200, or 300 nM cisplatin to the condition with no cisplatin added (0 nM). In the absence of T2AA, cisplatin had little effect on cell viability (ns = nonsignificant). T2AA treatment sensitized cells to cisplatin treatment (* p < 0.1, 90% confidence interval; ** p < 0.05, 95% confidence interval; *** p < 0.01, 99% confidence interval; **** p < 0.001, 99.9% confidence interval). F) Western blots of whole-cell lysates collected from PCNA-I1 or T2AA treated MRC-5 cells, or uninhibited (UI). Total protein was collected at 2, 4, 6, 8, and 24 hours post inhibitor addition. Blots were probed with α-PCNA or α-GAPDH antibody.

Before addressing the effects of PCNA inhibitors on HSV-1 infection, we first determined the concentrations at which PCNA inhibitors are cytotoxic to MRC-5 and Vero cells, which will be used for subsequent experiments. MRC-5 fibroblast or Vero cells were incubated in the presence of increasing concentrations of PCNA-I1 or T2AA for 24 hours before performing a CellTiter-Glo Luminescent Cell Viability Assay. The specific concentrations of PCNA-I1 [40–42,46,47] and T2AA [44,45,48–50] that were tested were based on previously published literature (S1 and S2 Tables). We incubated cells in the presence of the indicated inhibitor for 24 hours because all viral infection experiments were carried out for 24 hours or less. The CellTiter-Glo assay measures ATP levels and therefore the relative number of metabolically active cells. PCNA-I1 was not cytotoxic at and below 2.5 μM (Fig 2A) and T2AA was not cytotoxic at or below 12.5 μM (Fig 2B). Therefore, infection experiments were carried out with 2.5 μM PCNA-I1 or 12.5 μM T2AA.

**Fig 3:**
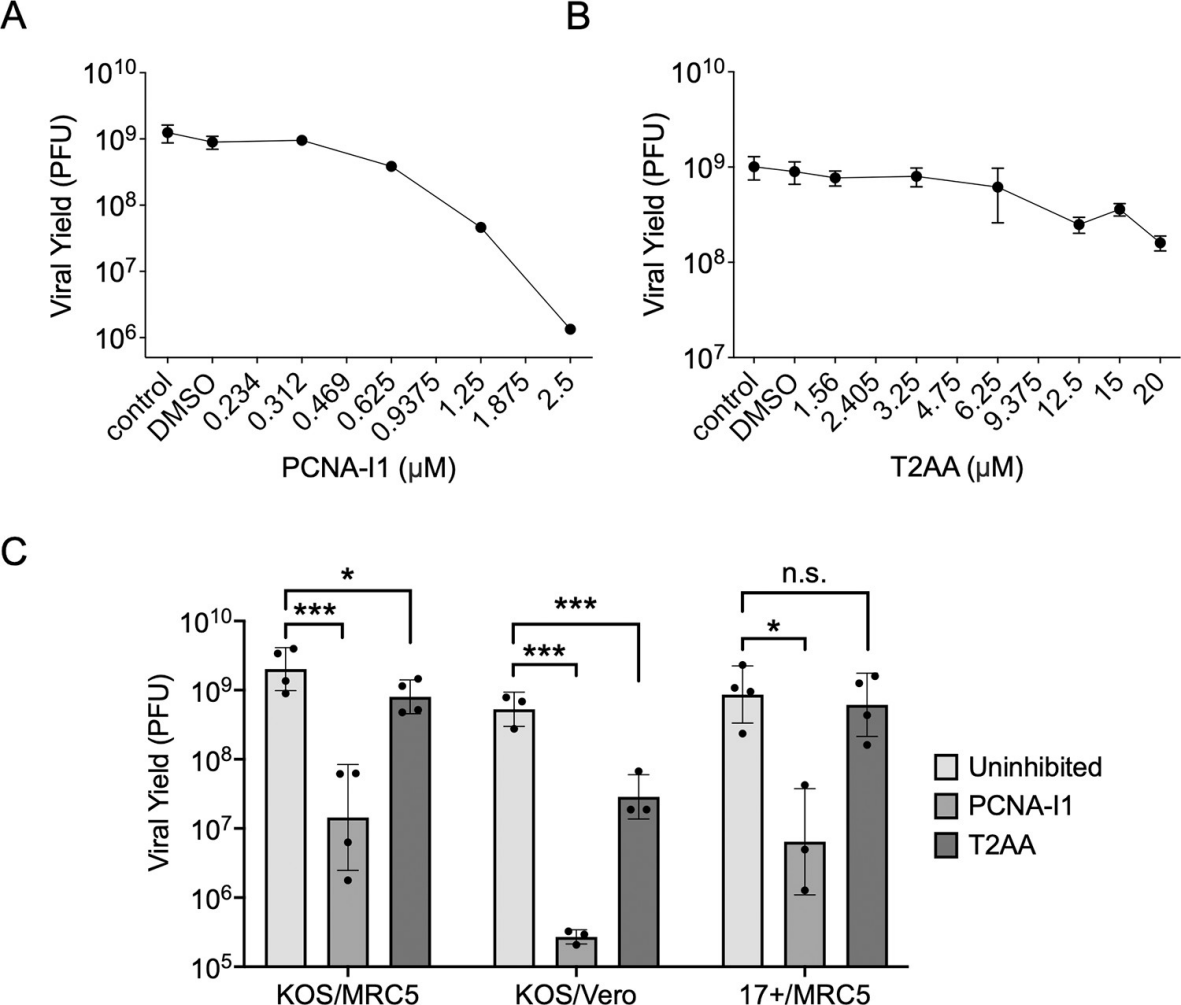
PCNA inhibitors inhibit HSV-1 infection. A-B) Effects of inhibitors on low multiplicity HSV-1 infection. Cells were supplemented with either PCNA-I1 (A) or T2AA (B), or as a control, uninhibited. Inhibitors were added one hour before and throughout infection. One million MRC-5 cells were infected at an MOI 0.1 PFU/cell in the presence or absence of PCNA-I1 or T2AA. Virus was collected 24 hours later. Viral yield was determined via plaque assay in Vero cells. All values represent the means of biological duplicate experiments with standard deviations. Data points without observable error bars represent highly reproducible data. C) Effects of inhibitors on high multiplicity infection. Experiments were conducted as described in (A-B) except infection was carried out at an MOI of 10 PFU/cell comparing two different lab strains (KOS and 17syn+) and cell types (MRC-5 and Vero cells). All data points represent independent biological replicates and error bars represent logarithmic standard deviations. One-way ANOVA with a Dunnett’s multiple comparisons test was performed to compare differences between inhibited and uninhibited groups (n.s. not significant, * p < 0.1, ** p < 0.05, *** p < 0.01).

We next determined if cellular DNA replication is inhibited when cells are treated with noncytotoxic concentrations of PCNA-I1 or T2AA. MRC-5 cells were seeded at a low density on coverslips to enrich for cells entering S phase. PCNA-I1 (2.5 μM) or T2AA (12.5 μM) was added to cells for 4 hours followed by 5-Ethynyl-2’-deoxycytidine (EdC) addition to the growth medium. EdC is an ethynyl modified nucleoside that is selectively incorporated into replicating DNA. Cells were incubated for two hours to enable EdC incorporation. Subsequently, cells were fixed and EdC-labeled DNA was tagged with a fluorophore by click chemistry, nuclei were stained with DAPI, and PCNA was detected by immunofluorescence. Ten images were captured in an unbiased manner with a 60X objective and the total number of nuclei and number of nuclei with EdC incorporated into the DNA were counted to determine the percentage of cells undergoing DNA replication under each experimental condition (Fig 2C and 2D). For the uninhibited control, 56.3% of cells incorporated EdC into replicating DNA. With T2AA treatment, 52.7% of cells incorporated EdC, whereas only 7% of cells incorporated EdC when treated with PCNA-I1. These data indicate that under noncytotoxic conditions, PCNA-I1 inhibits cellular DNA replication while T2AA has a modest effect. These differences are consistent with published results (S1 and S2 Tables) and likely because the drugs target different regions of the PCNA protein and act through different mechanisms. Previous studies have shown that PCNA-I1 inhibits DNA replication [40], while T2AA increases sensitivity of cells to DNA damaging agents [45]. To eliminate the possibility that PCNA-I1 or T2AA inhibition of cellular DNA replication will indirectly affect viral infection, all remaining experiments were carried out using confluent contact inhibited fibroblast cells that were experimentally verified to not be undergoing cellular DNA replication (with the exception of Fig 3C, Vero).

To ensure that T2AA is active at noncytotoxic concentrations, we treated cells in the presence of cisplatin, a chemotherapeutic drug that covalently attaches to purines and induces a DNA damage response, resulting in apoptosis [51]. Previous research indicates that T2AA alone does not inhibit cell growth and viability [45]. However, it does sensitize cancer cells to cisplatin, reducing HeLa and U2OS colony formation. To test whether T2AA sensitizes MRC-5 cells to cisplatin, cells were plated in the presence cisplatin only (100 nM, 200 nM, and 300 nM), T2AA only (12.5 μM), or both combined. After 24 hours, the CellTiter-Glo Luminescent Cell Viability Assay was performed. Cisplatin treatment of cells alone had a subtle effect on cell viability (Fig 2E). T2AA and cisplatin treatment together reduced cell viability up to 40%, indicating that T2AA does sensitize MRC-5 cells to DNA damaging agents and is therefore working as expected in our cell culture system.

We next determined the effects of PCNA inhibitors on PCNA expression. Neither inhibitor is predicted to alter PCNA protein levels. MRC-5 cells were incubated in the presence of PCNA-I1 or T2AA for 2, 4, 6, 8, or 24 hours, protein samples were collected at the designated time points, and analyzed by western blotting. Consistent with previous observations [40,45], PCNA-I1 and T2AA treatment had no effect on PCNA protein levels relative to a glyceraldehyde 3-phosphate dehydrogenase (GAPDH) loading control (Fig 2F). Taken together with cytotoxicity and DNA replication data, we established conditions for PCNA-I1 and T2AA treatment that are not cytotoxic to cells, do not alter PCNA protein levels in the cell, but effectively block cellular DNA replication (PCNA-I1) or DNA repair (T2AA).

### Treatment of cells with PCNA inhibitors results in reduced viral yield

We then investigated the effects of PCNA inhibition on the number of infectious virus particles that can be produced per cell. MRC-5 cells were seeded to confluency for 24 hours before they were infected with HSV-1 strain KOS at a multiplicity of infection (MOI) of 0.1 plaque forming units (PFU) per cell. Cells were inhibited with PCNA-I1 or T2AA one hour before and during infection. After 24-hours, virus was collected, and yield of infectious virus was determined by plaque assay in Vero cells. Treatment with PCNA-I1 resulted in a 1000-fold reduction in yield (Fig 3A), whereas T2AA inhibition resulted in a 5-fold reduction compared to an uninhibited control (Fig 3B). Therefore, both inhibitors cause a decrease in viral yield at noncytotoxic concentrations during low multiplicity infection of strain KOS in MRC-5 cells.

Additionally, to ensure that these trends are not unique to HSV-1 strain KOS, MRC-5 cells, or low multiplicity infection, we repeated the experiments in Fig 3A and 3B with different experimental conditions. To test if results are consistent during high multiplicity infection, MRC-5 cells were infected with KOS at an MOI of 10 PFU/cell in the presence or absence of inhibitors. Cells were inhibited one hour before and during infection with 2.5 μM PCNA-I1 or 12.5 μM T2AA. After 24-hours, virus was collected, and the number of infectious particles produced was determined by plaque assay in Vero cells (Fig 3C, KOS/MRC-5). As expected, the effects of inhibitors were reduced at high compared to low MOI. PCNA-I1 caused a 72-fold decrease in viral yield at high MOI compared to a 1000-fold decrease at low MOI and T2AA caused a 2.7-fold decrease at high MOI compared to a 5-fold decrease at low MOI. In addition, the effects of inhibitors on HSV-1 infection were assessed in Vero cells (Fig 3C, KOS/Vero). Regardless of cell type tested, PCNA-I1 and T2AA caused a decrease in viral yield after high MOI infection. However, the effects were exaggerated in Vero cells as PCNA-I1 caused a 2113-fold decrease in viral yield and T2AA caused a 16.7-fold decrease. Inhibitors had similar effects on high multiplicity infection of HSV-1 strain 17syn+ compared to KOS in MRC-5 cells, resulting in a 70.8-fold decrease in yield in the presence of PCNA-I1 and a 1.3-fold decrease in yield in the presence of T2AA (Fig 3C, 17+/MRC-5). Therefore, viral yield reduction due to PCNA inhibition is consistent across HSV-1 lab strains. Previously, PCNA targeting siRNAs were shown to cause a 100-fold decrease in viral yield when knockdown cells were infected at an MOI of 5 PFU/cell [36]. Our results demonstrate similar trends with the PCNA-I1 inhibitor, supporting that PCNA plays a role in HSV-1 infection.

For the remainder of the experiments, we investigated the effects of PCNA inhibitors on the HSV-1 infectious cycle using strain KOS and confluent MRC-5 cells. To enable investigation of single-step growth, from this point on, all infections were carried out at an MOI of 10 PFU/cell.

### PCNA-I1 reversibly inhibits HSV-1 DNA replication

**Fig 4:**
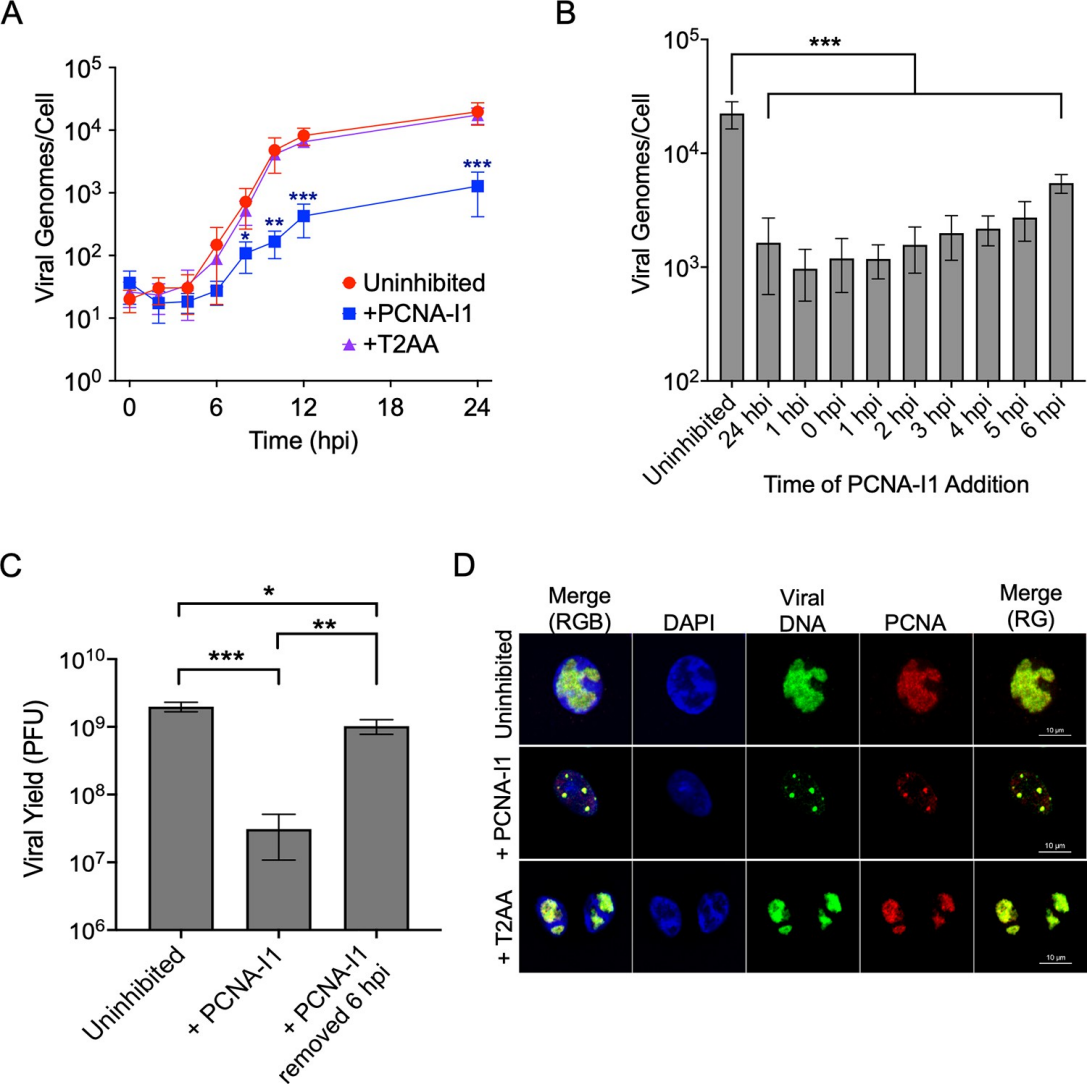
PCNA-I1 reversibly blocks HSV-1 DNA replication. A) Effects of PCNA-I1 and T2AA on HSV-1 DNA replication. MRC-5 cells were uninhibited or supplemented with PCNA-I1 (2.5 μM) or T2AA (12.5 μM) and infected with strain KOS at an MOI 10 PFU/cell. Total DNA was collected every 2 hours for 12 hours and at 24 hours post infection (hpi). The number of viral and cellular genomes were determined by qPCR relative to standard curves generated from purified viral or human DNA and the number of viral genomes per cell were determined. All values represent the means of biological triplicate experiments and error bars represent standard deviations. One-way ANOVA with a Dunnett’s test was performed to compare each time point of inhibited groups to the corresponding uninhibited control. Only statistically significant differences are shown (* p < 0.1, ** p < 0.05, *** p < 0.01). B) Effect of the time of PCNA-I1 addition on viral DNA replication. MRC-5 cells were supplemented with 2.5 μM PCNA-I1 either 24 or 1 hour before infection (hbi), at the time of infection, or at 1, 2, 3, 4, 5, or 6 hpi. Total DNA was collected at 12 hpi. Viral and cellular genome number were determined by qPCR as in (A). All values represent the mean of biological duplicate experiments and error bars represent standard deviation. One-way ANOVA with a Dunnett’s test was performed (* p < 0.1, ** p < 0.05, *** p < 0.01). C) Analysis of the reversibility of PCNA-I1 inhibition. MRC-5 cells were infected at an MOI 10 PFU/cell with strain KOS and were either uninhibited or supplemented with 2.5 μM PCNA-I1 one hour before and during infection (+PCNA-I1). In another sample, PCNA-I1 was removed at 6 hpi, cells were washed three times with TBS, and normal growth medium was replaced for the remainder of infection (+PCNA-I1 removed at 6 hpi). For all samples, virus was collected at 24 hpi. Viral yield was measured by plaque assay in Vero cells. All values represent the means of biological duplicate experiments with standard deviations. One-way ANOVA with Tukey’s multiple comparisons test was performed (* p < 0.1, ** p < 0.05, *** p < 0.01). D) PCNA is recruited to viral DNA despite PCNA inhibitor treatment. Vero cells were infected at an MOI 10 PFU/cell with strain KOS and were treated with 2.5 μM PCNA-I1 or 12.5 μM T2AA one hour before and during infection. EdC was incorporated into replicating viral DNA between 4–8 hpi and cells were fixed at 8 hpi. EdC labeled DNA was covalently attached to Alexa Fluor 488 (green) and PCNA was detected by immunofluorescence (red). Scale bars, 10 μM.

As PCNA is essential for cellular DNA replication and is found at viral replication forks in vivo, we predict that it is important for efficient HSV-1 DNA replication in cells. We therefore investigated the effects of PCNA-I1 and T2AA treatment on viral DNA replication (Fig 4A). To do this, MRC-5 cells were supplemented with PCNA-I1 or T2AA one hour before and during infection with KOS. Viral DNA was collected at two-hour intervals and the total viral genomes per cell were determined via quantitative real-time PCR (qPCR). The rate of viral DNA replication did not change in the presence of T2AA when compared to an uninhibited control. Treatment with PCNA-I1 resulted in a 15-fold reduction of viral DNA replication, with the rate most notably decreased between 4- and 8-hours post infection (hpi).

We then determined what effect the timing of PCNA-I1 addition has on HSV-1 DNA replication. MRC-5 cells were infected with HSV-1 strain KOS at an MOI of 10 PFU/cell. Treatment with PCNA-I1 occurred either 24 hours before infection, 1 hour before infection or at 1, 2, 3, 4, 5, or 6 hpi. Viral and cellular DNA were collected at 12 hpi and the total number of viral genomes/cell was determined by qPCR (Fig 4B). In the absence of PCNA-I1, approximately 22,000 viral genomes were produced per infected cell. PCNA-I1 had the greatest effect when added before the onset of viral DNA replication (before 3 hpi) causing ~15-fold reduction in the number of viral genomes per cell. After the onset of viral DNA replication, the effects of the timing of addition corresponded with the number of rounds of viral DNA replication that were allowed to occur before the addition of PCNA-I1. This experiment demonstrates specific inhibition of HSV-1 DNA replication by PCNA-I1.

Furthermore, to determine if inhibition of viral DNA replication by PCNA-I1 is reversible, PCNA-I1 was removed at 6 hpi and viral yield was determined at 24 hpi (Fig 4C). To investigate this, MRC-5 cells were infected at an MOI 10 PFU/cell and were inhibited with 2.5 μM PCNA-I1 one hour before and during infection. At 6 hpi, PCNA-I1 supplemented medium was removed and infected cells were washed three times with TBS to fully remove residual PCNA-I1. Fresh growth medium was added back to the cells. Virus was collected 24 hpi and infectious viral yield was determined by plaque assay in Vero cells. We found that the removal of PCNA-I1 restored infectious virus production to levels similar to an uninhibited control (Fig 4C). These data demonstrate that PCNA-I1 is a reversible inhibitor of HSV-1 DNA replication.

We next determined by immunofluorescence (IF) imaging if PCNA is localized to viral replication compartments during PCNA inhibition. Vero cells were plated on coverslips and infected with HSV-1 at an MOI of 10 PFU/cell. T2AA and PCNA-I1 treatment occurred one hour before and during infection. At 4 hpi, EdC was incorporated into viral DNA replication compartments for four hours before fixing. This was followed by click chemistry to tag EdC-labeled DNA with a fluorophore and immunofluorescence to detect the PCNA protein (Fig 4D). Consistent with previous observations, PCNA is recruited to replicating viral DNA during infection (no inhibitor) [20]. Inhibition with both T2AA and PCNA-I1 did not inhibit PCNA recruitment to viral replication compartments. However, treatment with PCNA-I1 resulted an overall reduced size of replication compartments consistent with a block in viral DNA replication.

### PCNA-I1 and T2AA treatment cause decreased HSV-1 late gene expression

**Fig 5:**
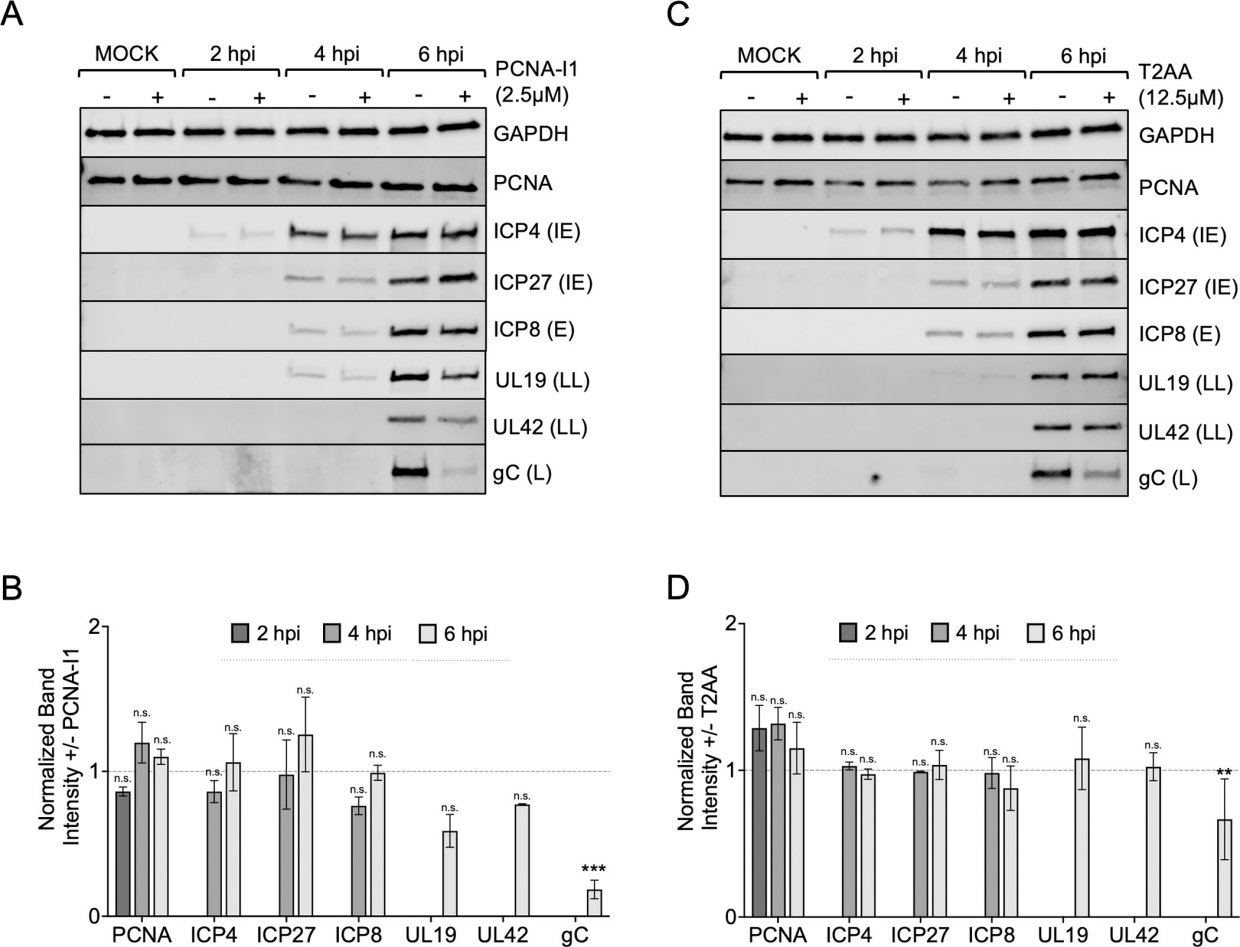
Effect of PCNA inhibitors on the temporal cascade of viral protein expression. A/C) Western blots of whole-cell lysates collected from cells infected or mock infected in the presence of PCNA-I1 (2.5 μM), T2AA (12.5 μM), or no inhibitor. MRC-5 cells were infected with HSV-1 strain KOS and total proteins were collected at 2, 4, and 6 hpi. Blots were probed with antibodies as indicated on the right. All infections were carried out at an MOI of 10 PFU/cell and all inhibitor treated cells were treated 1 hour before and throughout infection. B/D) Average fold change in protein expression (+inhibitor/-inhibitor) was determined and error bars represent standard deviations from biological duplicate experiments. Before calculating fold change, band intensities were normalized by dividing by the GAPDH signal detected from the same sample. Unpaired, two-tailed student’s t-tests were performed to compare the normalized band intensities of inhibited (+ PCNA-I1 or + T2AA) to uninhibited groups for each time point and each protein (* p < 0.1, ** p < 0.05, *** p < 0.01).

We next examined the effect of PCNA inhibitors on viral protein expression. MRC-5 cells were infected with HSV-1 strain KOS at an MOI of 10 PFU/cell in the presence of PCNA-I1 or T2AA. Whole cell lysates were collected at 2, 4, and 6 hpi and viral and cellular proteins were identified by Western blotting (Fig 5A/5C). Individual protein levels were normalized to GAPDH expression and fold change was determined by dividing the normalized band intensity in the presence of inhibitor by the normalized band intensity in the absence of the PCNA inhibitor (Fig 5B/5D). PCNA-I1 and T2AA treatment had no effect on immediate early (ICP4 and ICP27) or early (ICP8) viral protein expression. PCNA-I1 had a subtle effect on leaky late protein expression (UL42 and UL19), whereas T2AA treatment resulted in no change in leaky late protein expression. Interestingly, both PCNA-I1 and T2AA treatment resulted in a decrease in true late gene expression as indicated by reduced glycoprotein C (gC) expression. Taken together, neither inhibitor had an effect on viral immediate early or early protein expression, but did result in decreased late protein expression. These data are consistent with PCNA-I1 causing a block in viral DNA replication, as late gene expression is dependent on viral genome replication. On the other hand, these data suggest that T2AA affects subsequent steps in the infectious cycle, after the onset of viral DNA replication, that enable or contribute to late gene expression.

**Fig 6:**
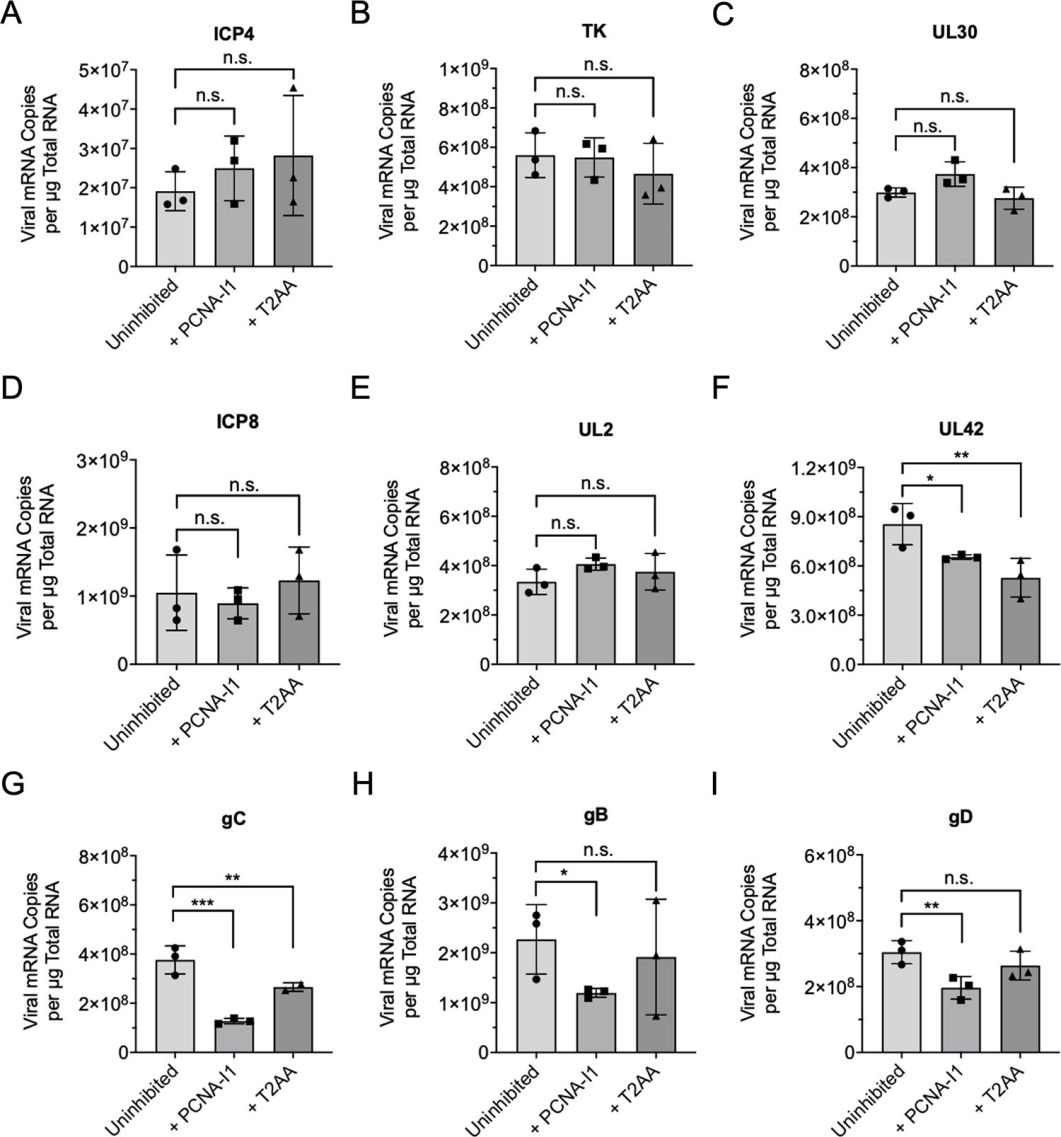
Effects of PCNA inhibitors on viral mRNA levels. MRC-5 cells were infected at an MOI 10 PFU/cell and supplemented with either PCNA-I1 (2.5 μM), T2AA (12.5 μM), or no inhibitor for one hour before and during infection. At 6hpi, total RNA was isolated, reverse transcribed, and amplified by qPCR. The number of viral mRNA copies per μg of total RNA was determined relative to a standard curve for each viral gene. A) ICP4 is a representative immediate early gene. B) TK, C) UL30, D) ICP8, and E) UL2 are representative early genes. F) UL42 is a representative leaky late gene. G) gC, H) gB, and I) gD are representative late genes. One-way ANOVA with a Dunnett’s multiple comparisons test was performed to compare inhibited groups to the corresponding uninhibited control (n.s. no significant difference, * p < 0.1, ** p < 0.05, *** p < 0.01).

To further investigate how PCNA inhibitors alter viral gene expression, we quantified the number of representative viral transcripts expressed when infection was carried out with and without PCNA-I1 and T2AA (Fig 6). MRC-5 cells were infected at an MOI 10 PFU/cell in the presence of PCNA-I1, T2AA, or no inhibitor. Total RNA was isolated at 6 hpi followed by reverse transcription and quantitative real-time PCR (RT-qPCR). Across all experimental groups, viral immediate early (ICP4) and early (TK, UL30, ICP8, UL2) mRNA levels did not change in the presence of PCNA inhibitors (Fig 6A–6E). These data are consistent with protein expression data in Fig 5. Levels of UL42 mRNA (leaky late) decreased in the presence of both PCNA-I1 and T2AA (Fig 6F). However, this effect was only apparent at the protein level during PCNA-I1 treatment (Fig 5). Viral late gene, gC, mRNA expression decreased in the presence of PCNA-I1 and T2AA (Fig 6G), which is consistent with decreased gC protein levels (Fig 5). Other viral late genes that were tested (gB, and gD) also consistently had decreased mRNA levels during PCNA-I1 treatment (Fig 6H and 6I). This indicates that the block in viral late gene expression is not exclusive to gC. However, levels of these late mRNAs did not decrease in the presence of T2AA compared to the uninhibited group. Together, these data are consistent with PCNA-I1 causing a defect in viral genome replication, resulting in reduced leaky late and late viral gene expression. In addition, T2AA has a much weaker and less consistent effect on late viral gene expression.

### PCNA-I1 and T2AA treatment result in increased production of defective HSV-1 virus particles

**Fig 7:**
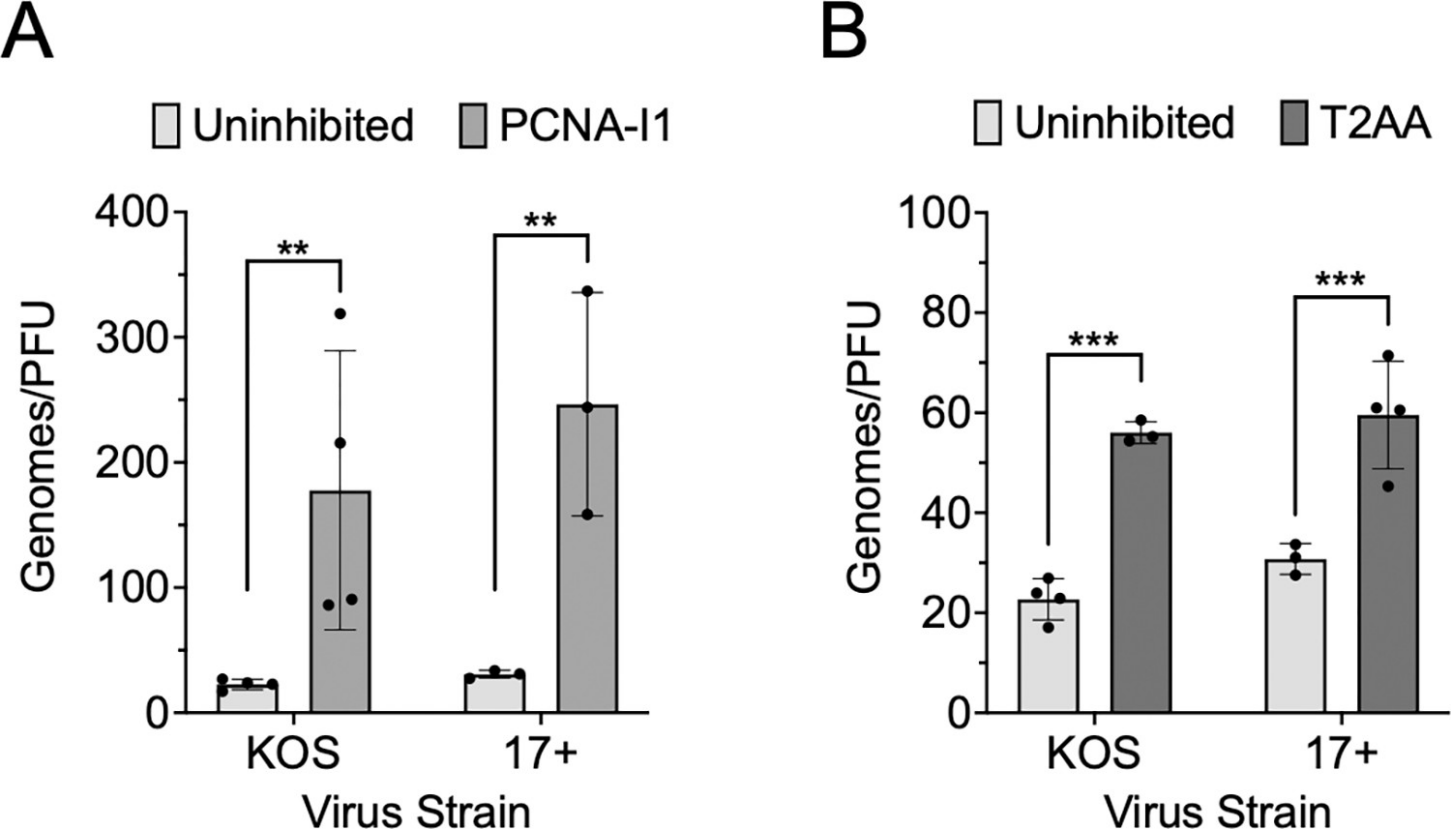
PCNA inhibitors cause decreased infectious virus production. Cells were supplemented with either A) PCNA-I1 or B) T2AA, or as a control, uninhibited. Inhibitors were added one hour before and throughout infection. One million MRC-5 cells were infected at an MOI 10 PFU/cell in the presence or absence of 2.5 μM PCNA-I1 or 12.5 μM T2AA. Virus was collected 24 hours later. PFU was measured via plaque assay on Vero cells (Fig 3). Viral genomes per PFU were quantified by isolating viral DNA from collected virus, followed by qPCR relative to a standard curve. Unpaired, two-way t-tests were performed to compare inhibited to uninhibited groups (* p < 0.1, ** p < 0.05, *** p < 0.01).

As shown in Fig 3C, PCNA-I1 resulted in a 72-fold decrease in HSV-1 yield compared to standard infection conditions (MOI 10 PFU/cell). However, results in Fig 4A demonstrated a 15-fold decrease in the total number of viral genomes per cell after PCNA-I1 inhibition. We also observe a decrease in glycoprotein expression, suggesting that a subset of genomes that are produced are packaged into capsids that are destined to be assembled into defective virus particles. Given this information, we next determined the genome/PFU ratio of virus produced in the presence of inhibitors compared to standard infection conditions. Viral DNA was isolated from the collected virus (Fig 3C) and the quantity of viral genomes in each sample were determined by qPCR (Fig 7). Under normal infection conditions, 22–29 viral genomes were detected per PFU. After PCNA-I1 treatment, there were ~8–10 fold more genomes detected per PFU (Fig 7A) and after T2AA inhibition there were 2.0–2.6 fold more genomes per PFU (Fig 7B). Taken together, treatment of cells with PCNA inhibitors increases the likelihood that defective virus particles will be produced. In addition, the increase in genomes per PFU may result from an accumulation of viral DNA mutations because of insufficient viral DNA repair during inhibitor treatment.

### PCNA inhibition alters protein recruitment to viral genomes

We next investigated how viral and cellular factors that associate with viral genomes change due to PCNA-I1 and T2AA treatment. MRC-5 cells that were plated to confluency and contact inhibited were infected with wild-type KOS at an MOI of 10 PFU/cell and either uninhibited or inhibited with 2.5 μM PCNA-I1 or 12.5 μM T2AA one hour before and during infection. Plating MRC-5 cells to confluency allows cells to enter G_0_ phase, ensuring that only replicating viral DNA is labeled and purified [20]. Cells were incubated in the presence of EdC from 4–6 hpi and nuclei were isolated at 6 hpi. EdC-labeled viral DNA was specifically and irreversibly tagged via covalent attachment of a biotin azide group. Tagged DNA was isolated with streptavidin-coated beads. Associated viral and/or cellular factors were then eluted and identified by mass spectrometry (MS). For each condition, a corresponding negative control was included to account for background binding to the streptavidin-coated beads. The control experiment was carried out in parallel, except that EdC was not added to the growth medium (-EdC). Factors were considered enriched on viral DNA if there were at least five spectral counts in the +EdC sample and at least four-fold more spectral counts in the +EdC sample compared to the -EdC control. Any protein that fit these criteria for two biological replicates of at least one experimental condition (KOS, KOS+T2AA, or KOS+PCNA-I1) was included for downstream analysis (S3 Table).

To account for common contaminants of MS datasets, we ran the protein list through the CRAPome database [52]. This database includes results from 716 affinity purification MS datasets and allows for the identification of proteins that may be isolated non-specifically. Proteins that were identified in 50% of affinity purification assays were considered potential contaminants and were removed from the lists. Of the proteins removed, 30% were ribosomal proteins, 6% were heat shock proteins, and 11% were heterogeneous nuclear ribonucleoproteins (hnRNPs).

**Fig 8:**
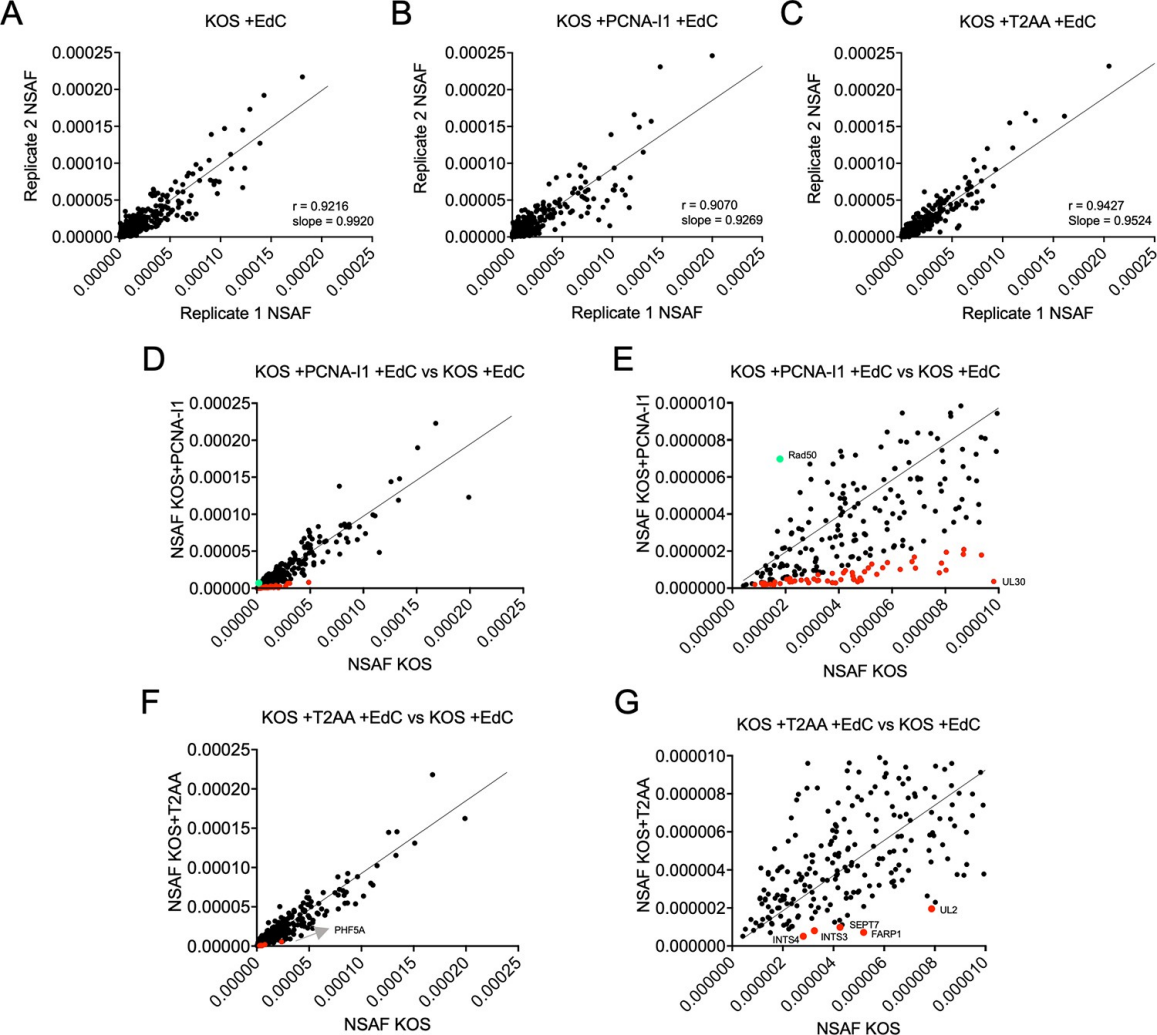
The use of NSAF to compare protein abundance in replicate viral aniPOND data sets and between different infection conditions. Comparison of the NSAF of viral and cellular proteins that associate with viral genomes in viral aniPOND assays at 6 hpi. Conditions include A) KOS +EdC, B) KOS + 2.5 μM PCNA-I1 +EdC, or C) KOS + 12.5 μM T2AA +EdC. Each point represents an individual protein with the NSAF of that protein from one biological replicate plotted on the x-axis and another biological replicate plotted on the y-axis. The linear regression line is shown with the slope. The r value represents the calculated Pearson correlation coefficient. (D) The average NSAF of individual proteins was calculated and plotted for KOS +EdC (x-axis) and KOS +PCNA-I1 +EdC (y-axis) aniPOND datasets. (E) The graph was generated as in (D) except that the axes were adjusted to highlight proteins that are more or less abundant in the presence of PCNA-I1. Proteins that had at least 4-fold decreased association with PCNA-I1 are presented in Fig 9B. (F) and (G) Graphs were generated as in (D) and (E) except that the y-axis represents the average NSAF of individual proteins found in aniPOND datasets in the presence of T2AA. Red data points represent proteins that decreased at least 4-fold and green data points increased at least 4-fold in the presence of either PCNA-I1 or T2AA.

To further analyze MS data, the spectral abundance factor (SAF) was determined by dividing the spectral counts (SpC) by the molecular weight of that protein. This accounted for any differences in protein size. We then calculated the normalized spectral abundance factor (NSAF) by dividing the SAF by the total SpC identified in an entire sample to account for any differences in overall protein yield and in the total amount of DNA isolated [53]. We plotted NSAF values between biological replicates of each experimental group and calculated the correlation (r) and linear regression. For each replicate dataset, there was a high reproducibility with r values >0.90 and slopes >0.92 (Fig 8A–8C). Therefore, NSAF of individual proteins between replicate experiments were reproducible.

In uninhibited groups, we identified that viral replication proteins (UL30, UL42, UL9, and ICP8/UL29), viral transcription regulatory factors (ICP4, ICP22, ICP27), and the viral uracil glycosylase (UL2), alkaline nuclease (UL12), and structural proteins enriched on replicated viral DNA at 6 hpi (S3 Table). In addition, cellular factors that are involved in cellular transcription, co-transcriptional RNA processing, modulation of chromatin, and DNA repair were also present. Furthermore, proteins involved in nucleic acid metabolism that may bind to EdC off of the viral genome were identified by aniPOND including UL50 (dUTPase), UL39 (ribonucleotide reductase—RIR1), and UL23 (thymidine kinase—TK). These data are consistent with previously published datasets [11,20–22].

The average NSAFs of biological replicates were used to compare KOS +EdC +PCNA-I1 to KOS +EdC (Fig 8D and 8E) and KOS +EdC +T2AA to KOS +EdC (Fig 8F and 8G). NSAF values that differed by more than four-fold when comparing inhibitor treated conditions to uninhibited controls were considered significantly less or more enriched on viral DNA in the presence of T2AA or PCNA-I1 [54]. Proteins that were more enriched are highlighted in green and less enriched are highlighted in red in Fig 8D–8G.

**Fig 9:**
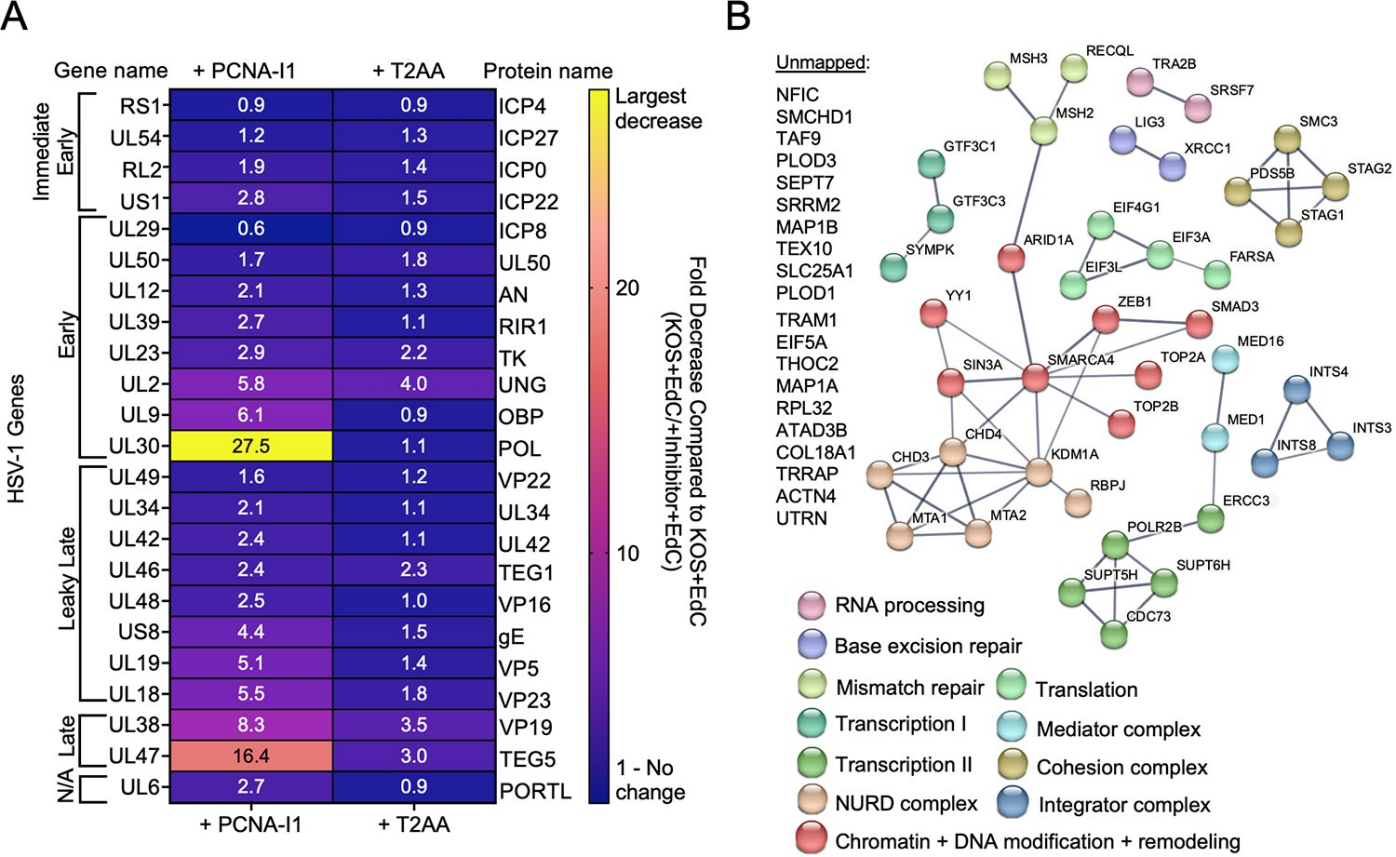
Viral and cellular proteins associated with replicating HSV-1 DNA change due to PCNA inhibition. A) Heat map indicating all viral proteins identified in viral aniPOND datasets and their fold change in abundance comparing PCNA-I1 (2.5 μM) or T2AA (12.5 μM) treated cells to an uninhibited control. HSV-1 genes are classified by their gene class (immediate early, early, leaky late, or late). Viral genes that are not classified are under “N/A”. Fold decrease was calculated as the NSAF of KOS+EdC divided by KOS +inhibitor +PCNA-1. Raw values indicate fold change and this is emphasized by the heat map. Values of less than 1.0 indicate an increase in protein association during inhibition. B) STRING diagram of human proteins that had at least a 4-fold decrease in association with viral DNA in the presence of 2.5 μM PCNA-I1. The STRING diagram shows predicted physical and functional interactions between human proteins. Biological process associated with identified proteins are labeled in varying colors. The unmapped list represents proteins that were not predicted to have high confidence protein-protein interactions with the other identified proteins using STRING. Some minor modifications were made to the STRING diagram to group known complex members together. ARID1A was originally grouped with ‘Mismatch repair proteins’ but was changed to be grouped with ‘Chromatin + DNA modification + remodeling.’ ERCC3 was originally grouped with the ‘Mediator complex’ but was changed to be grouped with ‘Transcription II’.

We next focused on comparing viral protein association with replicated viral DNA in the presence of inhibitors (Fig 9A). The levels of viral immediate early gene products associated with viral DNA did not change significantly in the presence of either inhibitor (inhibitor +EdC) compared to infection with KOS in the absence of inhibitors (KOS+EdC). Of note, for both PCNA-I1 and T2AA treated cells, the NSAF for ICP4 did not change compared to the untreated control (KOS +EdC/KOS +EdC +inhibitor = 0.9). ICP4 is the major viral transcription factor that binds to double stranded DNA during infection, is an immediate early gene product, and is essential for transcription initiation of early and late viral genes [7]. At the time viral DNA was isolated (6 hpi), ICP4 coats viral DNA in a sequence non-specific manner [12] and is therefore a good internal control and support for the use of NSAF to compare protein levels between datasets.

There was little to no difference in the association of HSV-1 early and leaky late gene products with viral genomes in the presence of T2AA (less than 2-fold). The one exception was the early viral gene product UL2 (uracil-DNA glycosylase), which decreased during T2AA treatment by 4-fold (Fig 9A). In addition, in the presence of T2AA, viral genome-associated late proteins (capsid and tegument) were less associated with replicated HSV-1 DNA (UL38 decreased by 3.5-fold and UL47 decreased by 3.0-fold), an observation that is consistent with T2AA treatment causing a decrease in late protein expression. When infection was carried out in the presence of T2AA, there was little to no change in viral genome association of most cellular factors when compared to an uninhibited group (Fig 8F and 8G). Host factors FERM RhoGEF and pleckstrin domain-containing protein 1 (FARP1), integrator complex subunit 4 (Ints4) and subunit 3 (Ints3), Septin-7, and the PHD finger-like domain containing protein 5A (PHF5A) decreased by at least 4-fold in the presence of T2AA (Fig 8F and 8G). All other cellular factors were recruited to viral DNA in similar abundances with T2AA as in uninhibited groups. Taken together, T2AA selectively inhibits the recruitment of select factors to replicated viral DNA including the viral base excision repair factor UL2 and cellular factors involved in transcription elongation (Ints3, Ints4, and PHF5A).

PCNA-I1 treatment affected the recruitment of specific viral and cellular factors with viral DNA (Fig 9). Levels of the single-stranded DNA binding protein ICP8 (UL29) associated with viral DNA increased slightly (KOS +EdC/KOS +PCNA-I1 +EdC = 0.6), while the viral origin binding protein UL9 (6.1-fold) and UL2 (5.8-fold) decreased in the presence of PCNA-I1 (Fig 9A). Across both biological replicates, UL30 was not stably associated with purified viral DNA given PCNA-I1 treatment, resulting in the greatest viral factor decrease of 27.5-fold (Fig 9A and 8E). These proteins are all products of early genes, of which expression is not affected by PCNA-I1 (Figs 5A/5B and 6B–6D). Additionally, there was a consistent decrease in association of viral leaky late and late gene products, consistent with the observation that there is a decrease in replication-dependent leaky-late and late protein and mRNA expression during PCNA-I1 treatment (Figs 5A/5C and 6E–6I).

There was a selective change in host factor recruitment to viral DNA during PCNA-I1 treatment. Only 15% of viral genome associated proteins decreased in the presence of PCNA-I1, suggesting that PCNA-I1 inhibition does not cause a general decrease in abundance of all proteins associated with viral DNA (S3 Table). STRING maps were created based on mass spectrometry data to outline the cellular proteins identified on viral DNA that decrease by at least 4-fold when infection was carried out in the presence of PCNA-I1 (Fig 9B). The most significant decrease was observed for the DNA repair protein RECQL (30.4-fold less +PCNA-I1). Additional DNA repair and maintenance proteins that decreased in the presence of PCNA-I1 include mismatch repair proteins (MSH2, MSH3), topoisomerases (TOP2A, TOP2B), and base (LIG3, XRCC1, as well as viral UL2) and nucleotide (XRCC3) excision repair proteins. Components of the cohesion complex (SMC1A, PDS5B, STAG2, SMC3, and STAG1) and factors involved in transcription regulation also decreased including an RNA polymerase II subunit (POLR2B), Integrator (INTS4, INTS3, INTS8) and Mediator (MED16, MED1) complex members, a transcription factor II D subunit (TAF6), transcription elongation factors (SPT5, SPT6, CDC73), the general transcription factor 3C polypeptides (GTF3C1, GTF3C3) and polyadenylation factor (SYMPK). In addition, factors involved in chromatin modification and remodeling (SIN3A, YY1, ZEB1, KDM1A/Lsd1) including Mi-2/NuRD (CHD3, CHD4, MTA1, MTA2) and Swi/Snf (ARID1A, SMARCA4) complex members decreased in abundance on replicated viral DNA in the presence of PCNA-I1. Interestingly, the cellular protein Rad50 was the only protein to increase in abundance on viral DNA by 4-fold in the presence of PCNA-I1 (Fig 8D/8E).

### MRN complex members are recruited to replicating viral DNA in the presence of PCNA-I1

**Fig 10:**
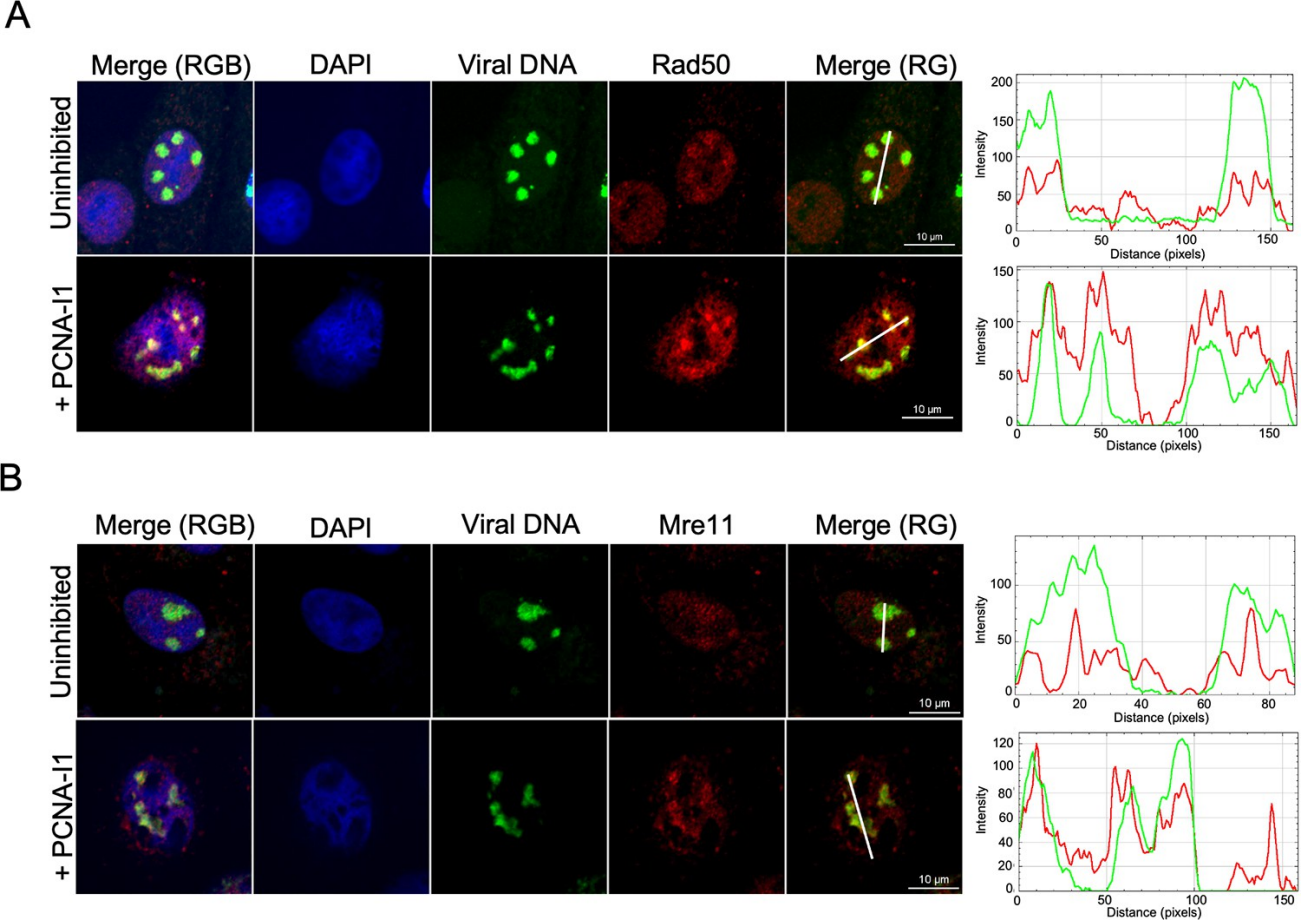
MRN complex members associate with replicating viral DNA during PCNA-I1 inhibition. Vero cells were infected at an MOI 10 PFU/cell with strain KOS and were either uninhibited or treated with 2.5 μM PCNA-I1 one hour before and during infection. EdC was incorporated into replicating viral DNA between 4–6 hpi and cells were fixed at 6 hpi. EdC labeled DNA was covalently attached to Alexa Fluor 488 (green) and A) Rad50 (GeneTex 13B3) or B) Mre11 (GeneTex 12D7) were detected by immunofluorescence (red). Scale bars, 10 μM. All images were taken using the same laser intensities. Traces were generated using the RGB profiler plugin in ImageJ and correspond to the white line drawn on the red/green merge (Merge (RG)) panel.

Because we observed an increase in Rad50 recruitment to replicated viral DNA when infection was carried out in the presence of PCNA-I1, we wanted to confirm these findings via immunofluorescence (Figs 10A and S1A). Rad50 is a component on the MRN complex. Another component of the MRN complex, Mre11 has been previously demonstrated to be associated with replicating HSV-1 DNA [55–57]. Therefore, we also probed for Mre11 (Figs 10B and S1B). Vero cells were plated on glass coverslips and infected with HSV-1 at an MOI of 10 PFU/cell. PCNA-I1 treatment occurred one hour before and during infection. Control cells were not treated with PCNA-I1. At 4 hpi, EdC was incorporated into viral DNA replication compartments for two hours before fixing. This was followed by click chemistry to tag EdC-labeled DNA with a fluorophore and immunofluorescence to detect protein recruitment (red) to replicated viral DNA (green). Consistent with aniPOND data, Rad50 was recruited to viral DNA in the presence and absence of PCNA-I1, with an apparent increase in recruitment to replication compartments when infection was carried out in the presence of PCNA-I1 (Figs 10A and S1A). RGB trace files generated show colocalization of red (Rad50) and green (viral DNA) traces in the presence and absence of PCNA-I1. However, there is consistently increased intensity of red traces with PCNA-I1 compared to uninhibited, indicating greater relative abundance during inhibitor treatment.

Consistently, we found that Mre11 was also recruited to viral DNA with a greater relative abundance during PCNA-I1 inhibition compared to an uninhibited control (Figs 10B and S1B). Mre11 RGB trace files indicate generalized staining of nuclei in uninhibited groups. PCNA-I1 inhibition resulted in more consistent overlap between viral DNA and Mre11 as indicated by RGB trace files. These data confirm that PCNA-I1 inhibition results in increased recruitment of MRN complex members to replicated viral DNA.

## Discussion

In this study, we demonstrate that PCNA inhibitors PCNA-I1 and T2AA inhibit HSV-1 infection. In a previous study, we demonstrated that PCNA associates with HSV-1 replication forks in a replication-dependent manner [21] and others have shown that PCNA knockdown results in reduced viral infection [36]. Here, we present novel observations that PCNA-I1 treatment results in a distinct block in UL30 recruitment to viral DNA and decreased HSV-1 DNA replication and replication-coupled processes. On the other hand, T2AA treatment blocks recruitment of base excision repair and transcription factors to viral DNA and results in reduced late gene expression and infectious virus production. These data indicate that PCNA is not only present at viral replication forks, but also provide insight into the functions of PCNA during viral DNA replication and coupled processes. Taken together, this study reveals new insight into the mechanism of HSV-1 DNA replication in vivo and the potential to target PCNA or viral or cellular factors that are recruited to viral DNA by PCNA for antiviral treatment. This study also highlights the use of viral aniPOND to compare protein recruitment to viral DNA during chemical inhibition of viral processes to provide mechanistic understanding of inhibitor function in vivo.

HSV-1 encodes seven core viral proteins that are sufficient to carry out DNA replication in vitro, including the viral processivity factor UL42 [18,19]. This raises the question as to why HSV-1 would utilize two processivity factors. UL42 is a unique processivity factor in that it forms a monomer in vivo [38,39] and therefore does not form a ring or clamp that slides along the double stranded DNA adjacent to the replication fork. Rather it is proposed to hop along the double stranded DNA while tethering UL30, the viral DNA polymerase [58]. Another difference between PCNA and UL42 is that PCNA is able to tether specific cellular proteins to replicating DNA including factors involved in chromatin remodeling [59–61], Okazaki fragment maturation [24], translesion synthesis [25–29], base [62,63] and nucleotide excision repair [30], homologous recombination [31], and mismatch repair [23,32–34]. We therefore predict that PCNA adds additional processivity to UL30 and/or forms a scaffold to recruit cellular factors to viral DNA during replication.

### Proteins enriched on replicating viral DNA

**Fig 11:**
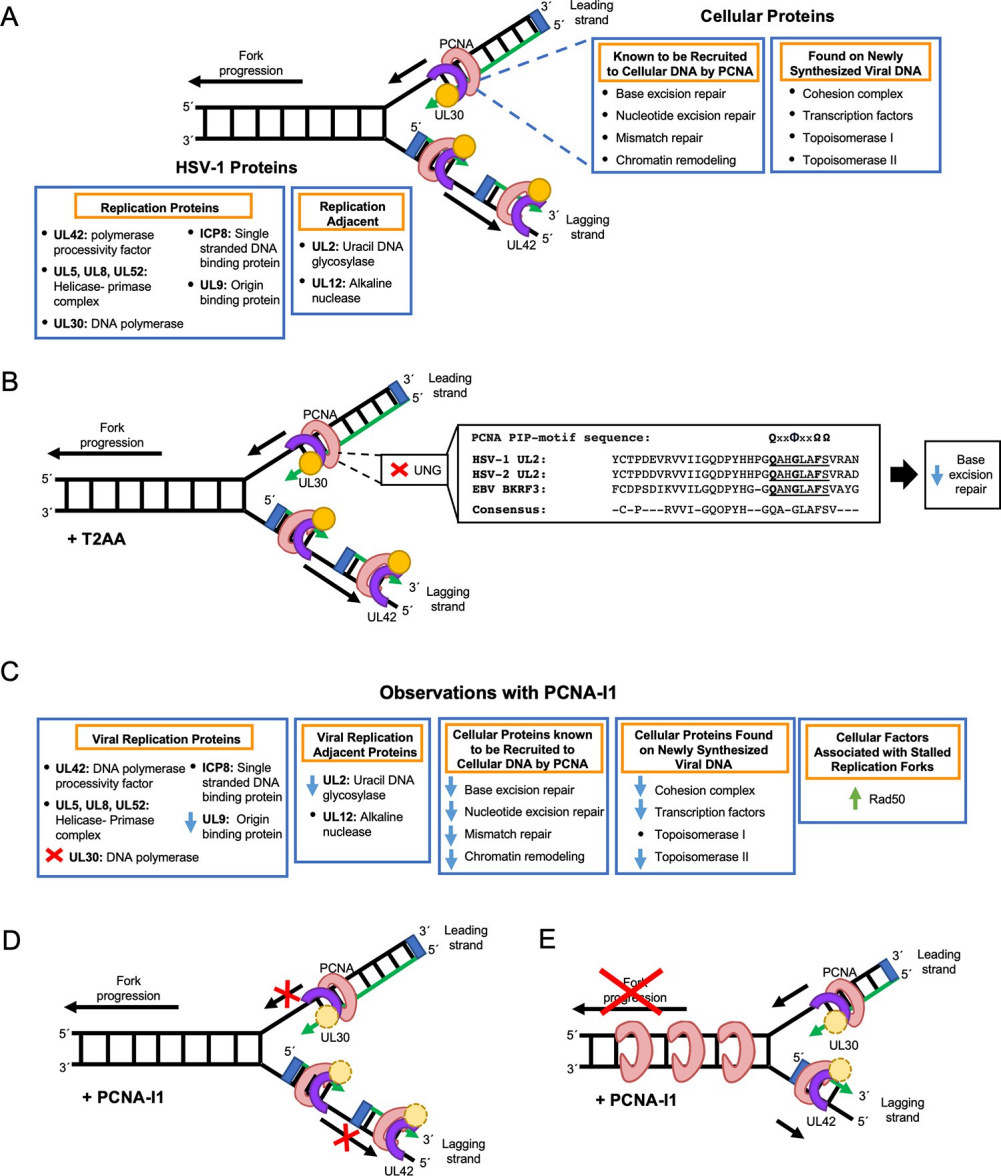
Model depicting the effects of PCNA inhibitors on HSV-1 DNA replication and repair. A) Model depicting proteins that associate with HSV-1 replication forks. PCNA is depicted in pink, UL30 in yellow, UL42 in purple, and the RNA primer in blue. Viral proteins are labeled to the left and cellular proteins are labeled on the right. B) T2AA is known to block PCNA protein-protein interactions via the PCNA IDCL (Fig 1D). Based on the data presented in this study, T2AA inhibition causes a defect in recruitment of UL2 to viral DNA. A PIP-like motif sequence is present in UL2, which is conserved in both the HSV-2 (UL2) and Epstein-Barr virus (EBV) (BKRF3) uracil DNA glycosylases (UNG). C) Proteins that are absent or significantly decreased on replicating viral DNA in the presence of PCNA-I1 are indicated with a red x or blue arrow, respectively. We present two models to explain these observations. D) Model 1: PCNA is unable to facilitate UL30 processivity and progression. E) Model 2 –During the second round of viral DNA replication, PCNA is unable to unload from the previously synthesized lagging DNA strand.

Viral aniPOND results demonstrate that during HSV-1 infection under uninhibited conditions, PCNA associates with replicating viral DNA, as well as the viral DNA replication machinery including the viral DNA polymerase UL30, processivity factor UL42, single stranded DNA binding protein ICP8, and origin binding protein UL9 (Fig 11A, S3 Table). Viral replication adjacent proteins were also present including the viral uracil glycosylase UL2 and alkaline nuclease UL12. In addition to viral factors that associate with replicating and replicated HSV-1 DNA, many cellular proteins were also enriched, including base and nucleotide excision repair, mismatch repair, and chromatin remodeling factors. These repair and chromatin modification factors have been found to be recruited to cellular DNA through interactions with PCNA. In addition, cellular proteins that are typically found on newly replicated viral DNA were also identified in the uninhibited control and include members of the cohesion complex, transcription factors, and topoisomerases. These data are consistent with previously published results using iPOND, aniPOND, and other approaches to identify HSV-1 genome associated proteins [20–22,56].

### T2AA treatment results in a defect in viral replication-coupled processes

The use of PCNA inhibitors that block specific regions of the PCNA protein provide mechanistic understanding of PCNA function during viral infection. T2AA blocks protein interactions between the PCNA IDCL and proteins containing the PIP-box peptide motif [43,44]. The PIP-box motif is a conserved PCNA-interaction motif consisting of eight residues including a glutamine at position 1, a hydrophobic amino acid at position 4, followed by an aromatic amino acid at position 7 and 8 [64]. Viral aniPOND revealed that when infection is carried out in the presence of T2AA, the amount of viral uracil glycosylase (UNG), UL2, that associates with replicating viral DNA decreased by 4-fold compared to an uninhibited control (Fig 8A). UNGs are DNA repair enzymes that initiate base excision repair by cleaving the N-glycosidic bond to remove uracil bases from DNA. It has previously been demonstrated that cellular UNG2 colocalizes with PCNA during cellular DNA replication to rapidly remove uracil shortly after misincorporation into replicated DNA [65]. Cellular UNG2 has a PIP-box motif, **Q**KT**L**YS**FF**, and has been shown to physically and functionally interact with PCNA to facilitate efficient base excision repair [63]. Interestingly, we identified a similar motif in HSV-1 UL2 at amino acids 185 to 192 (**Q**AH**G**LA**F**S) (Fig 11B). This PIP box-like motif is conserved between herpes viruses and is missing an aromatic amino acid at position 8. The PIP-motif is a loosely conserved sequence [64] and x-ray crystallography has revealed stable binding of PCNA to non-consensus PIP-motifs that lack the position 8 aromatic residue including DNA polymerase ι [66]. Data presented here, in combination with published findings described above, lead to the model that viral UL2 is recruited to replicating viral DNA through interactions between the UL2 PIP-like motif and cellular PCNA to couple viral DNA replication with base excision repair in vivo (Fig 11B).

Interestingly, UL2 is dispensable for HSV-1 production in tissue culture [67]. This is consistent with the minimal effect of T2AA on viral yield (Fig 3). However, there is an increase in noninfectious virus production (Fig 7B), consistent with a potential accumulation of viral genome mutations in the presence of T2AA.

We also found that T2AA inhibits late viral gene expression (Figs 5C/5D and 6). It is possible that the defect in UL2 recruitment can negatively impact late viral gene expression because of misincorporation of uracil into replicated viral DNA. On the other hand, it is possible that factors involved in the switch to activate transcription-coupled late gene transcription are in part recruited through interactions with the PCNA IDCL. In support of this, aniPOND results show a decrease in cellular Integrator complex members Ints4 (5.4-fold) and Ints3 (4.0-fold) recruited to replicated viral DNA (Fig 8G). Integrator is important for transcription elongation of transcripts produced by RNA polymerase II [68] and previous research has shown that the Integrator complex is enriched on replicating viral DNA [11,20]. Another transcription factor that decreased in abundance on replicating viral DNA during T2AA treatment was PHF5A (4.3-fold) (Fig 8F). PHF5A regulates release of promoter-proximal paused RNA polymerase II of PAF1 complex target genes [69]. Perhaps T2AA blocks recruitment of transcription elongation factors to replicated viral DNA, causing the decrease in transcription-coupled late viral gene expression (Figs 5 and 6, gC). The increase in noninfectious virus particle production observed during T2AA treatment (Fig 7B) is also consistent with a decrease in glycoprotein expression, resulting in the likely production of viruses that contain genomes but are defective for attachment and entry.

### PCNA-I1 treatment results in a defect in viral DNA replication

PCNA-I1 stabilizes the PCNA homotrimer and therefore likely affects PCNA loading, unloading, or sliding along the DNA [40,41]. Here we show that PCNA-I1 does not inhibit PCNA recruitment to viral DNA (S3 Table) or early steps in infection but does block viral DNA synthesis (Fig 4A) and subsequent late viral gene expression (Figs 5A/5B and 6). Viral aniPOND revealed that PCNA-I1 treatment results in a reduction in the association of the viral DNA polymerase UL30, origin binding protein UL9, and UL2 with viral DNA (Figs 8E and 9A). In addition, there was a general decrease in association of cellular proteins involved in DNA repair, genome architecture, chromatin modification and organization, and transcription regulation and an increase in Rad50 binding during PCNA-I1 inhibition (Figs 8D/8E and 10A). Rad50 is one of the first proteins recruited to collapsed replication forks and these results may be indicative of fork stalling leading to fork collapse in the presence of PCNA-I1.

One outstanding question is how PCNA is loaded onto viral DNA. During cellular DNA replication, PCNA-I1 decreases PCNA association with DNA by stabilizing the PCNA trimer, potentially inhibiting PCNA loading [40]. However, when added before infection, PCNA-I1 does not impact PCNA levels on viral DNA, indicating that it can still be loaded onto viral DNA (S3 Table). In the absence of PCNA-I1, multiple subunits of replication factor C (RFC), a five-subunit clamp loader complex, were identified to be associated with viral DNA by aniPOND, although they were below the confidence threshold (S3 Table–Replicate 1 Raw Data and Replicate 2 Raw Data). In the presence of PCNA-I1, RFC was not captured by aniPOND of replicated viral DNA. Note that aniPOND uses native conditions for purification of EdC-labeled DNA and may not capture transient or dynamic interactions. Perhaps during PCNA-I1 treatment, HSV-1 utilizes an alternative mechanism for PCNA loading or at the time the DNA is isolated, RFC is no longer associated. Further studies are required to fully characterize the mechanisms of PCNA loading (and unloading) from HSV-1 DNA.

The data presented in this study suggest a few potential models by which PCNA-I1 may affect HSV-1 DNA replication (Fig 11C–11E). Note that these models are not mutually exclusive and observed effects on viral DNA replication may be a result of a combination of these models.

### Model 1: PCNA-I1 inhibits UL30 processivity

All proteins and complexes that decrease in abundance on viral DNA in the presence of PCNA-I1 are recruited to viral DNA in a replication-dependent manner (Fig 11C) [11,21]. Therefore, it is reasonable to speculate that PCNA-I1 treatment affects viral DNA polymerase processivity. UL30 is one of the few viral proteins that is completely absent from viral aniPOND datasets during PCNA-I1 treatment (S3 Table, Figs 8E and 9A). Compared to an uninhibited control, UL30 abundance on viral DNA decreased by an average of 27.5-fold (Fig 9A). Perhaps, PCNA-I1 inhibits the ability of PCNA to effectively tether UL30 to replicating viral DNA, preventing replication fork progression and replication-coupled association of other viral and cellular factors with viral DNA. Consistent with a processivity defect, the rate of viral DNA replication is significantly reduced during PCNA-I1 treatment. In addition, the viral origin binding protein UL9 decreased in abundance on viral DNA by 6.1-fold, indicative of a defect in new origin firing during PCNA-I1 treatment. Cellular DNA repair proteins that decreased in the presence of PCNA-I1 include mismatch repair proteins (RECQL, MSH2, MSH3), base excision repair proteins (UL2, LIG3, XRCC1), and a nucleotide excision repair factor (XRCC3) (Fig 9B). All of these repair mechanisms have been found to involve protein recruitment by PCNA to damaged cellular DNA [23], thereby coordinating DNA replication with repair [70]. Topoisomerase proteins TOP2A and 2B also decrease, likely because of decreased helicase unwinding and replication fork progression. In addition, there is a decrease in cohesion complex association in the presence of PCNA-I1. During cellular DNA replication, this complex works to hold replicated sister chromatids together during S phase, allowing for homology-based DNA recombination to occur [71]. Furthermore, cellular transcription factors decrease in abundance including Integrator and Mediator complex members and proteins involved in the regulation of transcription elongation. As late gene expression is dependent on viral DNA replication, it is possible that the decrease in transcription factors associated with viral DNA (Fig 9B) and late gene product expression (Figs 5A/5B and 6) is a direct result of the observed viral DNA replication defect. Taken together, one model predicts that PCNA-I1 alters UL30 processivity, leading to a stalled/slow replication fork, decreased DNA repair, and downstream defects in late viral gene expression (Fig 11D).

In a previous study, it was concluded that PCNA knockdown affects histone deposition on HSV-1 DNA [36]. Consistent with this observation, we also noted a decrease in Swi/Snf and NuRD complex member association with viral DNA during PCNA-I1 inhibition. This result may provide insight into the mechanism by which histone protein deposition is regulated on replicated HSV-1 DNA.

Data presented here demonstrate that there is an increase in components of the host MRN complex associated with viral DNA during PCNA-I1 treatment (Figs 8D/8E and 10). The MRN complex is comprised of Mre11, Rad50, and Nbs1. This complex is first to detect and bind to DNA damage to coordinate a DNA damage response at DNA double strand breaks and collapsed replication forks. Therefore, slowed replication due to a processivity defect of UL30 during PCNA-I1 inhibition may lead to replication fork stalling and collapse and increased recruitment of the MRN complex to viral DNA. MRN may play a role in stalled viral replication fork restart, perhaps through homologous recombination.

### Model 2: PCNA is unable to unload from HSV-1 DNA, acting as a replication fork barrier during the second round of DNA replication on the lagging strand

On the lagging strand, in order to effectively facilitate Okazaki fragment synthesis, PCNA must continuously load and unload from the DNA. By aniPOND, we found that PCNA abundance does not change on HSV-1 DNA during PCNA-I1 treatment compared to an uninhibited control (S3 Table) and IF data demonstrates that PCNA is still recruited to viral replication compartments during PCNA-I1 treatment (Fig 4D). Given that PCNA-I1 is thought to lock together PCNA monomers in the trimer form, it is possible that these observations are consistent with a PCNA unloading defect. Therefore, if PCNA is unable to unload from Okazaki fragments on the lagging strand, there would then be an accumulation of PCNA, obstructing a second round of DNA replication on this strand (Fig 11E). This could provide an alternative explanation for the slowed replication rate observed in Fig 4A if only the leading strand can undergo additional rounds of replication during PCNA-I1 treatment.

Replication fork barriers can cause replication proteins to dissociate from replicating DNA unless recombination restarts DNA synthesis [72]. This could provide an alternative explanation for the decrease in UL30 associated with viral DNA. If the replisome dissociates, this can produce a collapsed fork, which may result in a DNA double strand break. Our data indicate an increased accumulation of components of the MRN complex, perhaps beginning restart of collapsed forks though homologous recombination. A replication fork barrier would also block new origin firing, depending on the location relative to the origin. Consistent with this, we observed decreased viral origin binding protein, UL9 in the PCNA-I1 treated aniPOND dataset (Fig 9A).

### Model 3: PCNA-I1 inhibits a replication protein other than PCNA

There is always the possibility that PCNA-I1 may target a viral protein that we haven’t considered. One possibility is the viral DNA processivity factor UL42. However, binding assays followed by native gel electrophoresis and immunoblotting revealed that PCNA-I1 binds selectively to PCNA in the trimer form and stabilizes the trimer [40]. UL42 is structurally similar to PCNA but does not form multimers [39]. However, it is possible that PCNA-I1 causes UL42 to multimerize, thereby altering its ability to function on viral DNA. Additionally, we cannot rule out the possibility that UL42 and PCNA interact.

Another consideration is that PCNA-I1 binds to a structurally similar host protein. Further analysis specificity looked at the effect of PCNA-I1 binding to the 9-1-1 protein complex [40]. The 9-1-1 protein complex is a member of the clamp family proteins that also encircles DNA. Rad9, Rad1, and Hus1 form a trimer and together, assist in DNA repair [73]. PCNA-I1 does not bind to or stabilize the 9-1-1 trimer, indicating that it binds selectively to PCNA trimers [40]. In addition, we did not identify the 9-1-1 complex to be associated with viral DNA in this or previous studies. Therefore, although this is a possibility, it is probably not likely.

Additionally, T2AA and PCNA-I1 cause overlapping effects on viral infection. For example, both inhibitors result in a late gene expression (Figs 5 and 6G–6I) and virion assembly defect (Fig 7). aniPOND also showed a decrease in UL2 and Integrator association across both inhibitors. Similar phenotypes as a result of PCNA inhibition further support that these inhibitors are targeting PCNA rather than another viral or cellular protein.

## Conclusion

Here we used inhibitors that target different functions of PCNA to understand how a host protein is mechanistically involved in HSV-1 infection. Using a combination of approaches, including viral aniPOND, we identified how these inhibitors alter the virus life cycle and protein recruitment to replicated/replicating viral DNA, allowing us to generate models as to how the inhibitors facilitate specific blocks in the infectious cycle. Together with the findings that PCNA selectively associates with viral replication forks [21] and that PCNA knockdown by transfected siRNAs results in reduced viral infection (36), data presented here provide additional support for a role of PCNA in viral DNA replication. Furthermore, proposed models open new doors to further dissect how PCNA protein-protein interactions facilitate viral DNA replication and late gene expression. PCNA inhibitors are currently being investigated as anti-cancer therapies due to the preferential inhibitory effects on proliferating tumor cells that are actively engaged in DNA replication [46,74]. Our data signify that PCNA inhibitors or inhibitors of proteins recruited to viral DNA by PCNA have the potential to be adapted for antiviral treatment against HSV-1 infection.

## Materials and methods

### Cells and viruses

MRC-5 (human diploid lung fibroblast) and Vero (African green monkey kidney) cells were obtained from and propagated as recommended by ATCC. Either HSV-1 strain KOS or strain 17 syn+ was used for all infections.

### CellTiter-Glo luminescent cell viability assay

The CellTiter-Glo Luminescent Cell Viability Assay (Promega) was carried out to measure the effects of PCNA-I1 (Sigma Aldrich SML0730) and T2AA (Abcam ab146970) on cell viability. PCNA-I1 and T2AA were dissolved in DMSO to a final concentration of 50 mM and 100 mM respectively as stock solutions. Cells were plated in 96 well plates (1.25x10^4^ MRC-5 cells/well or 4x10^4^ Vero cells/well) for 24 hours prior to addition of inhibitors. Stock concentrations of PCNA-I1 and T2AA were diluted to the indicated concentrations. Diluted inhibitors were added and cells were incubated for 24 hours before conducting the CellTiter-Glo Luminescent Cell Viability Assay according to the manufacturer’s protocol. Luminescence was measured on a SpectraMax Plate Reader. Percent viability was measured as the relative light units (RLU) of each experimental condition divided by the untreated (cells only) control.

### Immunofluorescence and imaging of replicated DNA

To determine if PCNA inhibitors inhibit cellular DNA replication (Fig 2C), MRC-5 cells were seeded at 1.67x10^5^ cells/well in a 12-well dish on glass coverslips. 2.5 μM PCNA-I1 or 12.5 μM T2AA was added to cells for 4 hours. At 4h, EdC was incorporated into replicating cellular DNA until fixing at 6h. To determine if PCNA, Rad50, and Mre11 are recruited to replicating viral DNA in the presence and absence of inhibitors (Figs 4D, 10 and S1), a total of 1.67x10^5^ Vero cells were grown on glass coverslips and infected with KOS at an MOI of 10 PFU/cell in the presence or absence of 2.5 μM PCNA-I1 or 12.5 μM T2AA. Inhibitors were added one hour before and during infection. EdC labeling of viral replication compartments occurred between 4–6 hpi (Figs 10 and S1) or 4–8 hpi (Fig 4D). Cells were fixed with paraformaldehyde and click chemistry and immunofluorescence were conducted as previously described [20,75]. Primary antibodies included α-PCNA (PC-10, Abcam ab29), α-Mre11 (GeneTex, 12D7), and α-Rad50 (GeneTex 13B3). Images were taken with a Nikon Eclipse Ti2 Inverted Confocal Microscope. Trace files in Figs 10 and S1 were generated using the RGB profiler plugin in ImageJ.

### Cisplatin assays

The CellTiter-Glo Luminescent Cell Viability Assay (Promega) was carried out to measure the effects of cisplatin (Millipore Sigma PHR1624) on cell viability in the presence of T2AA. Cisplatin was prepared according to previously published literature [45] in 0.9% (w/v) aqueous sodium chloride to a final stock concentration of 5 mM. MRC-5 cells were plated in 96 well plates (1.25x10^4^ MRC-5 cells/well), diluted cisplatin and/or T2AA were added to cells as indicated, and cells were incubated for 24 hours before conducting the CellTiter-Glo Luminescent Cell Viability Assay. Luminescence was measured on a SpectraMax Plate Reader. Percent viability was measured as the RLU of each experimental condition divided by the untreated control.

### Western blotting

MRC-5 cells were plated to confluency in a 6 well dish (1x10^6^ cells/well). Cells were treated one hour prior and during infection with 2.5 μM PCNA-I1 or 12.5 μM T2AA. Cells were uninfected or infected with KOS at an MOI of 10 PFU/cell. Proteins were isolated from cells using Laemmli SDS sample buffer at indicated times. Western blotting was carried out using the following primary antibodies: α-ICP4 (Abcam ab6514), α-ICP8 (Abcam ab20194), α-gC (GICR1104), α-ICP27 (Abcam ab53480), α-VP5/ICP5 (Abcam ab6508), α-UL42 (Abcam ab19311-100), α-PCNA (PC-10, Abcam ab29), α-GAPDH (Invitrogen AM4300). Gel band intensity was quantified using the GelAnalyzer plugin in ImageJ and were normalized to GAPDH detected from the same sample.

### Viral yield assay

MRC-5 or Vero cells were seeded at a density of 1x10^6^ cells/well (MRC-5) or 5x10^5^ cells/well (Vero) in a 6-well dish and infected with strain KOS or 17 syn+ at an MOI of 0.1 or 10 PFU/cell. Cells were incubated with 2.5 μM PCNA-I1, 12.5 μM T2AA, or no inhibitor one hour before and during infection. After a 1-hour adsorption period, the inoculum was removed and cells were rinsed with tris buffered saline. Infected cells were incubated in medium containing the indicated concentration of PCNA-I1, T2AA, or no inhibitor. At indicated times, infected cells were collected by scraping into growth medium and freeze-thawed 3 times followed by sonication to release cell associated virus. Viral yield was determined by plaque assay in Vero cells.

### Viral DNA replication curve

MRC-5 cells were plated to confluency (1x10^6^ cells/well) in a 6-well dish. Cells were incubated with 2.5 μM PCNA-I1 or 12.5 μM T2AA one hour before and during infection. Cells were infected with KOS at an MOI of 10 PFU/cell. At the indicated time, DNA was isolated using DNA extraction buffer (0.5% SDS, 400 μg/ml proteinase K, 100 mM NaCl). Genome numbers were determined by real-time PCR relative to a standard curve generated from purified KOS DNA. Primers specific for the gC gene (gcf 5’GTGACGTTTGCCTGGTTCCTGG-3’, gcr 5’-GCACGACTCCTGGGCCGTAACG-3’) were used for viral DNA amplification. The number of cells per condition were determined by amplification of cellular DNA using primers specific for GAPDH (GAPDHf 5´- CAGAACATCATCCCTGCCTCTACT-3´, GAPDHr 5´-GCCAGTGAGCTTCCCGTTCA -3´) relative to a standard curve generated from purified human DNA.

### Time of PCNA-I1 addition assay

MRC-5 cells were seeded at 5x10^5^ cells/well in a 12-well dish. Cells were infected with KOS at an MOI of 10 PFU/cell. Cells were treated with 2.5 μM PCNA-I1 supplemented medium at indicated times before or after infection. At 12 hpi, DNA was isolated using DNA extraction buffer (0.5% SDS, 400 μg/ml proteinase K, 100 mM NaCl) followed by phenol/chloroform extraction. Viral and cellular genome numbers were determined by qPCR relative to a standard curve generated from purified viral or human DNA as indicated above.

### PCNA-I1 removal assay

MRC-5 cells were seeded at 1x10^6^ cells/well in a 6-well dish. Cells were infected with KOS at an MOI of 10 PFU/cell. Cells were treated with 2.5 μM PCNA-I1 supplemented medium one hour before and during infection. At 6 hpi, PCNA-I1 supplemented media was removed, cells were washed with tris buffered saline and fresh DMEM plus 10% FBS was added back to the wells. Infected cells were collected by scraping into growth medium at 24 hpi and freeze-thawed 3 times followed by sonication to release cell associated virus. Viral yield was determined by plaque assay in Vero cells.

### Viral mRNA extraction and RT-qPCR

MRC-5 cells were plated at 1x10^6^ cells/well in a 6-well dish. Cells were supplemented with either PCNA-I1 (2.5 μM) or T2AA (12.5 μM) one hour before and during infection. Cells were infected with KOS at an MOI 10 PFU/cell. At 6 hpi, total RNA was isolated using TRIzol Reagent (Invitrogen) followed by reverse transcription using a MMLV HP reverse transcriptase (Thermofisher) and Oligo dt(20) primer (Thermofisher). Viral genes were amplified using real-time PCR using the following primers: ICP4 (ICP4f 5’-CCACGGGCCGCTTCAC-3’, ICP4r 5’-GCGATAGCGCGCGTAGAA-3’), TK (TKf 5’- CCAAAGAGGTGCGGGAGTTT-3’, TKr 5’-ACCCGCTTAACAGCGTCAACA-3’), UL30 (UL30f 5’-CATCACCGACCCGGAGAGGGAC-3’, UL30r 5’-GGGCCAGGCGCTTGTTGGTGTA-3’), ICP8 (ICP8f 5′-CATCAGCTGCTCCACCTCGCG-3′, ICP8r 5′GCAGTACGTGGACCAGGCGGT-3′), UL2 (UL2f 5’-GACTTGCGTTTAGCGTGCGC-3′, UL2r 5’-CAACCGTGGCCGCTCATC-3′), UL42 (UL42f 5’-ACGTCCGACGGCGAGG-3’, UL42r 5’-CAGGCGCAACTGAACGTC-3’), gC (gCf 5’-GTGACGTTTGCCTGGTTCCTGG-3’, gCr 5’-GCACGACTCCTGGGCCGTAACG-3’), gB (gBf 5’-TACTGCGGCTGGCCCACCTTG-3’, gBr 5’-GCTCTCGCGCGTGGACCTG-3’), and gD (gDf 5′-CTATGACAGCTTCAGCGCCGTCAG-3′, gDr 5′-CGTCCAGTCGTTTATCTTCACGAGC-3′) [76]. The number of viral mRNA copies per μg RNA was calculated relative to standard curves generated using purified KOS viral DNA.

### Calculating viral genomes/PFU

Virus that was collected as described under “Viral Yield” was subjected to a DNA extraction. Virus was thawed on ice and diluted 1:100. An equal volume of 2X sodium dodecyl sulfate (SDS) sodium bicarbonate buffer (10% SDS, 7.5% sodium bicarbonate) was added to each sample followed by incubation overnight at 65°C. DNA samples underwent a phenol-chloroform extraction and genome numbers were determined by real-time PCR relative to a standard curve generated from purified viral DNA. Primers specific for the gC gene were used for viral DNA amplification. Genome/PFU was calculated by diving the calculated viral DNA by the total number of plaque forming units found in “Viral Yield”.

### aniPOND

aniPOND was carried out as previously described [75,77]. MRC-5 cells were plated to confluency (~7x10^7^ cells) in 600cm^2^ dishes. Cells were inhibited with either 2.5 μM PCNA-I1 or 12.5 μM T2AA one hour before infection and infected with wild-type KOS at an MOI 10 PFU/cell for one hour at room temperature. Inoculum was removed and cells were washed with TBS followed by the addition of fresh DMEM plus 10% FBS supplemented with either PCNA-I1 or T2AA. Cells were incubated at 37°C for four hours before addition of EdC at a final concentration of 25 μM followed by an additional two-hour incubation. The negative control for each sample was carried out in the absence of EdC. Nuclear extraction, click chemistry, cell lysis, and sonication were carried out as described previously except cell lysis occurred for 30 minutes on ice followed by sonication 8 times at 40% amplitude with a 3 mm probe. Proteins were eluted from streptavidin-coated beads by boiling in 2X SDS Laemmli sample buffer.

### Mass spectrometry and data analysis

Mass spectrometry was carried out by MSBioworks as previously described [20]. Proteins were considered enriched by aniPOND based on the following criteria: 1) protein had at least 5 SpCs in one experimental condition per data set, 2) protein was not detected in the negative control or was enriched by at least four-fold based on dividing SpC values, and 3) protein was detected in biological duplicate experiments. The spectral abundance factor (SAF) was determined for each protein. SAF is calculated by dividing the SpC by the molecular weight of that protein to account for differences in protein size. Normalized spectral abundance factor (NSAF) was calculated by dividing the SAF by the total SpC identified in that sample [53]. This accounts for differences in overall protein yield between samples and variation in total amount of DNA isolated.

For creating the STRING analysis, string-db.org was used [78]. Human gene names were used for a multi-protein search. Basic settings included: network type was full STRING network and network edges were set to confidence. Active interaction sources included text-mining, neighborhood, experiments, gene fusion, and co-occurrence. The minimum interaction score was set to 0.7 to predict high confidence interactions. Clustering options are MCL clustering with the inflation parameter of 3. Disconnected nodes were hidden from the string diagram but are included in a list adjacent to the STRING diagram (Fig 8B).

### Statistical analysis

All statistical analyses were performed using Graphpad Prism v9. The statistical test used for each figure are indicated in the figure legends. Biological replicates are plotted to each corresponding experimental condition. Each biological replicate consisted of the average of 2–3 technical replicates.

## Supporting information

S1 Table Concentrations of PCNA-I1 and cell lines used in previously published literature. (XLSX)

S2 Table Concentrations of T2AA and cell lines used in previously published literature. (XLSX)

S3 Table Summary of unprocessed and processed aniPOND mass spectrometry data. (XLSX)

S1 Fig Additional images of Rad50 and Mre11 colocalization with replicating viral DNA during PCNA-I1 inhibition. Vero cells were treated with either 2.5 μM PCNA-I1 or were uninhibited for one hour before and during infection. Cells were infected at an MOI 10 PFU/cell. EdC incorporation into replicating viral DNA occurred between 4-6hpi. EdC labeled DNA was covalently attached to Alexa Fluor 488 (green) and A) Rad50 (GeneTex 13B3) or B) Mre11 (GeneTex 12D7) were detected by immunofluorescence (red). Scale bars, 10 μM. All images were taken using the same laser intensities as Fig 10. Intensity traces were generated using the RGB profiler plugin in ImageJ and correspond to the white line drawn on the red/green merge (Merge (RG)) panel. Each panel includes two supporting images of each experimental condition in addition to Fig 10.
(TIF)
