# Supplementary figures and images for: Proliferating cell nuclear antigen inhibitors block distinct stages of herpes simplex virus infection
Source: PLoS Pathog. 2023 Jul 24;19(7):e1011539. doi: 10.1371/journal.ppat.1011539 (PMC10399828; doi:10.1371/journal.ppat.1011539)

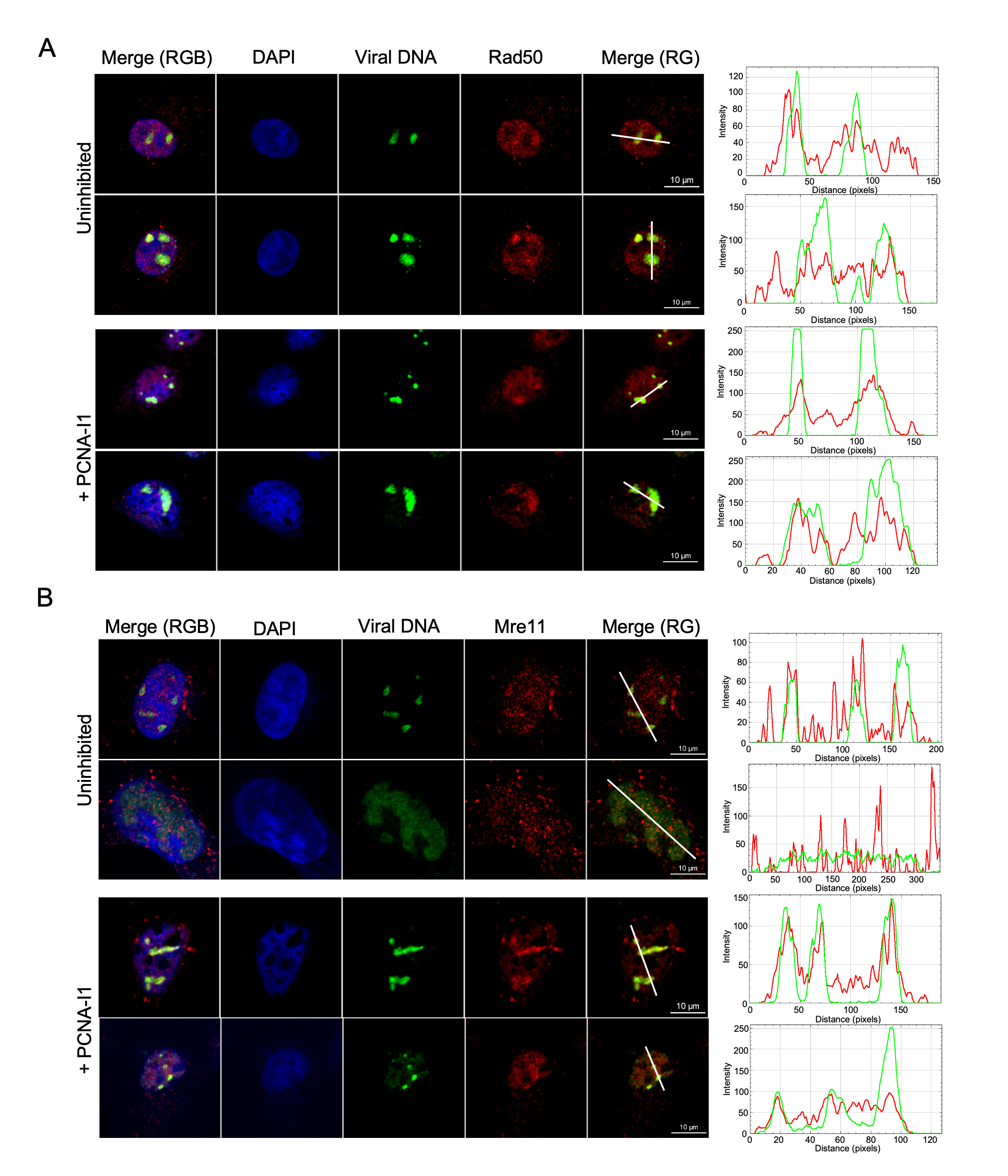

Supplement: S1 Fig — Vero cells were treated with either 2.5 μM PCNA-I1 or were uninhibited for one hour before and during infection. Cells were infected at an MOI 10 PFU/cell. EdC incorporation into replicating viral DNA occurred between 4-6hpi. EdC labeled DNA was covalently attached to Alexa Fluor 488 (green) and A) Rad50 (GeneTex 13B3) or B) Mre11 (GeneTex 12D7) were detected by immunofluorescence (red). Scale bars, 10 μM. All images were taken using the same laser intensities as Fig 10. Intensity traces were generated using the RGB profiler plugin in ImageJ and correspond to the white line drawn on the red/green merge (Merge (RG)) panel. Each panel includes two supporting images of each experimental condition in addition to Fig 10. (TIF) [file ppat.1011539.s004.tif]
